# Correction of eIF4E overactivation rescues translatome imbalance and core ASD-like behaviors in valproic acid-induced offspring mice

**DOI:** 10.1038/s41380-026-03517-3

**Published:** 2026-03-07

**Authors:** Miaoqi Huang, Han Ye, Yong Xu, Jiaoyan Xie, Xinyu Wang, Yan Luo, Peng Liu, Xuanyue Ma, Shiqing Zhang, Bin Jiang, Wen-Cai Ye, Yinghui Peng, Lei Shi

**Affiliations:** 1 State Key Laboratory of Bioactive Molecules and Druggability Assessment, Guangdong Basic Research Center of Excellence for Natural Bioactive Molecules and Discovery of Innovative Drugs, Guangzhou, 510632 China; 2 JNU-HKUST Joint Laboratory for Neuroscience and Innovative Drug Research, Guangdong Province Key Laboratory of Pharmacodynamic Constituents of TCM and New Drugs Research, Guangdong Hong Kong-Macau Joint Laboratory for Pharmacodynamic Constituents of TCM and New Drugs Research, College of Pharmacy, Jinan University, Guangzhou, 510632 Guangdong China; 3 Medical College, Jiaying University, Meizhou, 514031 Guangdong China; 4 Guangdong Province Key Laboratory of Brain Function and Disease, School of Medicine, Sun Yat-sen University, Shenzhen, 518107 China

**Keywords:** Autism spectrum disorders, Neuroscience

## Abstract

Perturbed protein synthesis plays a crucial role in the pathogenesis of autism spectrum disorder (ASD), but the altered translational pattern and underlying mechanism remain poorly understood. Here, we identified an exaggeration of global protein synthesis in the cerebral cortex of offspring mice following prenatal exposure of valproic acid (VPA), a well-established ASD model. Integrative analysis of polyribosome-based translatome and proteome data revealed remarkable upregulation of ribosomal and mitochondrial genes in VPA-exposed cortex at both translational and protein levels, but not transcriptional levels. Further analysis pinpoints that overactivation of the translation initiation factor eIF4E causes the aberrant translatome and mitochondrial impairments in VPA-exposed cortex. Pharmacological inhibition of eIF4E phosphorylation during juvenile displayed persistent effectiveness in mitigating ASD-like social deficits and stereotyped behavior in VPA mice until adulthood. Collectively, these findings demonstrate that eIF4E overactivation leads to imbalanced protein synthesis that favors translation of ribosomal and mitochondrial genes, causing core ASD-like behaviors.

## Introduction

Autism spectrum disorder (ASD) is a complex neurodevelopmental condition with heterogeneous clinical manifestations, characterized by social deficits and stereotyped behaviors as the core symptoms. The molecular pathophysiology of ASD involves a complex and poorly understood interplay between genetic and environmental factors, and effective interventions remain limited. Accumulating evidence indicates that aberrant mRNA translation, leading to perturbed proteostasis within different compartments of neurons especially the synapses, plays a key role in the pathogenesis of ASD [1–3]. The dysregulated protein synthesis may result from alterations of the translational regulatory proteins as well as the ribosomal proteins, both of which are among the key functional modules of high-confidence ASD candidate genes [4]. One well-known example is FMRP (fragile X messenger ribonucleoprotein protein), an mRNA-binding protein that in general negatively regulates protein translation, and its loss-of-function leads to elevated brain protein synthesis in both patient-derived cells and animal models [5]. The mammalian target of rapamycin complex 1 (mTORC1)-dependent translational regulatory pathway has gained notable attention as multiple components within this signaling are encoded by ASD risk genes, and an overall hyperactivation of the signaling is associated with ASD [6]. Moreover, several eukaryotic translation initiation factors (eIFs) and elongation factors (eEFs) are candidate genes for ASD. Their dysfunction can directly lead to neuronal translational abnormalities and ASD-like behaviors in mice, as observed in the cases with the cap-binding eIF4E and eIF4G [7–9].

The cap-binding protein eIF4E is the limiting component of the eIF4F initiation complex (eIF4E/eIF4A/eIF4G) and plays a central role in translation initiation, which is the rate-determining step of protein synthesis [10]. eIF4E serves as a pivotal node converging diverse signaling inputs to regulate mRNA translation. While functional changes of eIF4E have been reported across various neuropsychiatric disorders, including schizophrenia, major depressive disorder, and bipolar disorder, overactivation of eIF4E appears to be particularly prominent in the pathogenesis of ASD [11]. *EIF4E* is a high-confidence ASD risk gene, with its rare gain-of-function variants and copy number gains identified in individuals with syndromic ASD [12–15]. Functionally, eIF4E overexpression or hyperphosphorylation drives excessive neuronal translation and behavioral deficits characteristic of ASD [7, 8, 16–19]. In contrast, reduced eIF4E phosphorylation induces depression and anxiety-like behaviors [20, 21]. Importantly, hyperphosphorylation or increased expression of eIF4E is observed in multiple monogenic ASD models including *fragile X messenger ribonucleoprotein 1* (*Fmr1*) KO mice and *activity-dependent neuroprotective protein* (*Adnp*)^+/-^ mice, as well as in maternal immune activation (MIA)-induced ASD offspring [22–25]. Similar eIF4E dysregulation is also recapitulated in patient-derived neural progenitor cells of tuberous sclerosis complex (TSC), a rare genetic disorder strongly associated with ASD [26]. Pharmacological or genetic downregulation of eIF4E exhibits notable effects in reversing ASD-like behavioral deficits in some of these models [22, 27–30]. However, it remains unclear how eIF4E overactivation induces translatomic alterations, and whether these changes are commonly manifested in ASD models caused by polygenic and environmental risk factors.

Epidemiological surveys indicate that environmental factors contribute substantially to 40–50% of ASD cases [31, 32]. Among them, taking medications during pregnancy, such as the antiepileptic drug valproic acid (VPA), has been identified as the most well-established environmental contributor to ASD [33]. Children with prenatal exposure to VPA show developmental abnormalities and core autistic traits including social skills deficits and stereotyped behaviors [34]. These clinical ASD manifestations are robustly recapitulated in offspring of multiple species, including zebrafish, rodents, and non-human primates, after VPA is administered systemically to pregnant dams during early gestation period [35, 36]. Prenatal VPA exposure disrupts brain and physical development of offspring, and the animals exhibit core ASD-like symptoms such as social interaction deficits, impaired communication, repetitive behaviors, and cognitive rigidity, which closely mimic the DSM-5 criteria of ASD [35–37]. Moreover, the offspring show full-range of neuropathophysiological features recapitulating those of ASD children, including impaired neurogenesis, dendritic spine morphogenesis, and synaptic connectivity [37, 38]. Therefore, this model has a notable advantage over monogenic models for studying classic ASD cases that originate from prenatal insults. For the past three decades, it has been the most widely-used model induced by environmental factors to study the pathological mechanisms and develop therapeutic strategies of ASD [35, 37, 38].

Previous reports have revealed altered mTORC1 activity in the prefrontal cortex and hippocampus of VPA-exposed offspring [39–43]. However, direct evidence remains lacking regarding how protein synthesis is altered in this model and whether VPA affects certain ASD-associated factors, such as eIF4E, to modulate protein synthesis. Moreover, existing studies have been more focused on transcriptomic analysis of the VPA mice, with translatome unexplored. Given that the immediate expression of proteins is often not captured by transcriptional changes but is more closely associated with translational levels, analyzing the translatome to pinpoint key functional gene clusters affected by in utero VPA exposure will provide new evidence for understanding the protein synthesis perturbation theory of ASD.

In the current study, we identified exaggeration of protein synthesis in the cerebral cortex of juvenile VPA-exposed offspring. By integrative analysis of the transcriptome, polyribosome (polysome) profiling-based translatome, and synaptosomal proteome, we identified remarkable upregulation of ribosomal and mitochondrial genes at both translational and protein levels, but not transcriptional levels. Correspondingly, VPA mice exhibited higher cortical ATP production and more mitochondrial numbers within the cortical synapses. Molecular analysis revealed upregulation of several translation regulatory molecules that converge on the downstream overactivation of eIF4E in the cortex of VPA offspring. Introducing a phosphor-mimetic form of eIF4E in cultured cells recapitulated the translational alteration and mitochondrial dysfunction observed in VPA cortices. Finally, treatment with eFT508, an inhibitor of the eIF4E kinase MAPK-interacting kinase (MNK), effectively restored global protein synthesis and translation of mitochondrial and ribosomal mRNAs, concomitantly with corrected mitochondrial morphology and function. More importantly, administration of eFT508 during juvenile exhibited long-term effects to alleviate ASD-like behaviors in the VPA-exposed offspring. Collectively, these findings demonstrate that overactivation of eIF4E leads to imbalanced protein synthesis that favors translation of ribosomal and mitochondrial genes, causing core ASD-like behaviors. These results strengthen the key role of dysregulated protein synthesis in ASD pathology and highlight eIF4E as a promising target for therapeutic strategy development.

## Results

### VPA-induced ASD mice exhibit exaggerated cortical de novo protein synthesis and imbalanced translatome

VPA-induced offspring ASD mouse model was achieved by a single injection of VPA (500 mg/kg) to the pregnant mice on gestational day 12.5. Both male and female offspring from the same litters showed delayed eye opening and reduced body weight gain, with males displaying more pronounced developmental delay (Fig. S1a–d). Core ASD-like behaviors were also more prominent in male VPA offspring, who showed failures in social discrimination test and social approach/novelty impairments in Three-chamber test (Fig. S1e–n). Although female VPA offspring also showed reduced social abilities in the above tests, the deficits were much milder than males (Fig. S1e–n). Regarding the repetitive behavior, male VPA offspring exhibited marked stereotyped self-grooming, which was absent in female VPA offspring (Fig. S1o, p). These sex-dependent differences are also similarly reported by others [44–46]. General locomotor activity in the Open-field test was comparable between VPA and control groups in both sexes (Fig. S1q, r). Given that male offspring of in utero VPA exposure manifest both core behavior features of ASD, i.e., social deficits and stereotypy, we only studied males in the following experiments unless otherwise stated. In addition to these core behaviors, male VPA-exposed mice exhibited heightened anxiety level, indicated by the reduced head-dip times in the elevated Zero-maze test (Fig. S2a), whereas performed normal in various learning and memory tasks (Fig. S2b–e).

To evaluate whether in utero VPA exposure affects protein synthesis of the developing brain, we measured de novo global protein synthesis in the cerebral cortex at embryonic day (E)18.5, postnatal day (P)12, and P35 using L-azidohomoalanine (AHA)-based bioorthogonal non-canonical amino acid tagging (BONCAT) and puromycylation assays. Interestingly, the overall protein synthesis was not altered at both E18.5 and P12, but was significantly elevated in the cortex of VPA group at P35 (Fig. 1a–c). No change of hippocampal protein synthesis was observed in the VPA group (Fig. S3a). Polysome profiling was further employed to evaluate protein synthesis and distribution of ribosome components in the cortex at different stages. Consistent with the BONCAT and puromycylation results, the contents of 60S ribosome subunit, 80S monosome, and polysome were unaltered in the cortex of VPA group at E18.5 and P12; whereas at P35, the VPA-exposed cortex showed a decrease trend in peak areas of 60S and 80S and an increase in polysome peak area, resulting in significantly higher peak area ratio of polysome to the sum of 60S and 80S (Figs. 1d, S3b–d), indicative of elevated global protein synthesis.

**Fig. 1:**
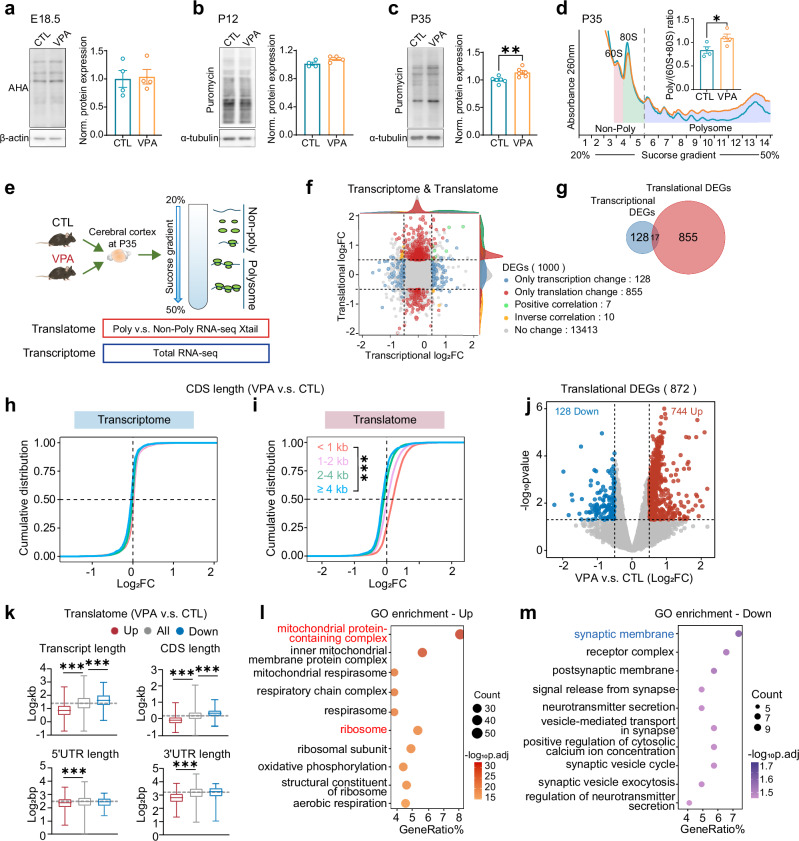
VPA mice exhibit exaggerated de novo protein synthesis and imbalanced translatome in the cerebral cortex. **a** Representative immunoblots (left panel) and quantification analysis (right panel) of L-azidohomoalanine (AHA)-labeled de novo global protein synthesis in cerebral cortex of control (CTL) and VPA mice at embryonic day (E) 18.5. β-actin was used as a loading control. *n* = 4 mice for both groups. Unpaired *t* test. **b,** **c** Representative immunoblots (left panels) and quantification analysis (right panels) of puromycin-labeled de novo global protein synthesis in cerebral cortex slices of CTL and VPA mice at postnatal day (P) 12 (**b**) and P35 (**c**). α-tubulin was used as a loading control. P12: *n* = 4 mice in each group. P35: *n* = 6 mice in each group. Unpaired *t* test, **p < 0.01. **d** Representative polysome profiling from the cerebral cortex of CTL and VPA mice at P35. The polysome/(60S + 80S) ratio (embedded panel) was calculated as the ratio of polysome peak area over the sum of 60S and 80S peak areas from each profiling, correspondingly. *n* = 4 mice in each group. Unpaired *t* test, *p < 0.05. **e** Experimental schematic of translatome and transcriptome obtained from the cerebral cortex of VPA and CTL groups at P35. **f** Log_2_ fold changes (Log_2_FC) of mRNAs (VPA vs. CTL) at transcriptional and translational levels. **g** Venn diagram showing little overlap of DEGs at both transcriptional and translational levels, and more DEGs at translational level. **h,** **i** mRNAs were classified into four populations by the following coding sequence (CDS) lengths: < 1 kb, 1–2 kb, 2–3 kb, and ≥ 4 kb. The Log_2_FC (VPA vs. CTL) of each length population in the transcriptome (left panel) and translatome (right panel) was shown as cumulative distribution curves. Two-sided KS test, < 1 kb vs. > 4 kb, D = 0.50559, ***p < 0.001. **j** Volcano plot showing translational DEGs of VPA vs. CTL groups. **k** Box-whisker Plot showing the entire transcript length, CDS length, 5’UTR length, and 3’UTR length of translational upregulated DEGs and downregulated DEGs, in comparison with those of all genes in the translatome. Kruskal-Wallis test, ***p < 0.001. The dotted line represents the median value of all genes. **l,** **m** Bubble plots showing Gene ontology (GO) enrichment result of translational up-regulated (**l**) and down-regulated (**m**) DEGs (VPA vs. CTL). Data are expressed as mean ± SEM.

To simultaneously analyze gene expression profiles at the transcriptional and translational levels, we conducted bulk RNA sequencing in parallel with polysome profiling-based RNA sequencing. mRNAs were separately extracted and sequenced from the total homogenate, translationally inactive non-polysome (non-poly) fraction, and translationally active polysome (poly) fraction of the cortical tissues from control and VPA-exposed mice at P35 (Fig. 1e). Principal component analysis (PCA) revealed a distinct separation of non-poly, poly, and total mRNA datasets, indicating a distinguishable mRNA abundance pattern in each population (Fig. S4a). Importantly, compared to the total mRNA datasets, the poly mRNA datasets showed more obvious separation between VPA and control groups (Fig. S4b, c). To obtain translatome information from each sample, the Xtail translation efficiency algorithm was adapted to quantify the value of each gene from the paired poly and non-poly data [47]. Transcriptome data were obtained from bulk RNA sequencing. A comparison between the translatome and the transcriptome revealed 145 transcriptional differentially expressed genes (DEGs) and 872 translational DEGs (|Log_2_FoldChange (Log_2_FC)| > 0.5 and *p*-value < 0.05), with only 17 shared genes (Fig. 1f, g, Table S1). This finding suggests that in utero VPA exposure distinctly affects postnatal gene expression profiles at the transcriptional and translational levels, and the alteration of translatome is more pronounced.

Since translation efficiency is highly dependent on transcript length, including the lengths of 5’ untranslated region (UTR), the coding sequence (CDS), and 3’UTR of mRNAs, we investigated whether VPA differentially affects the translation of transcripts with varying lengths. We categorized the mRNAs into four groups based on their CDS lengths: < 1 kb, 1–2 kb, 2–4 kb, and ≥ 4 kb, and analyzed the expression changes of each group in both the transcriptome and translatome. There were no obvious expression changes of transcripts at all lengths in the transcriptome, suggested by the cumulative distributions which reached 50% at Log_2_FC = 0 (Fig. 1h, i). By contrast, bidirectional changes in a length-dependent manner were observed in the translatome data, that is, shorter transcripts were more upregulated whereas longer ones more downregulated in the VPA-exposed cortex, and the cumulative distribution curve of CDS < 1 kb transcripts was significantly right-shifted than that of CDS ≥ 4 kb transcripts (Fig. 1i). Further analysis of the translational DEGs identified 744 upregulated and 128 downregulated genes (Fig. 1j). Notably, the average lengths of the whole sequence, CDS, 5’UTR, and 3’UTR of upregulated transcripts were all remarkably shorter when compared to those of entire population of transcripts (Fig. 1k). Conversely, the average lengths of the whole sequence and CDS of downregulated transcripts were longer than those of the entire population (Fig. 1k). We further showed that the poly(A) tail lengths, according to a published dataset [48], were similar in both translationally upregulated and downregulated genes in VPA group compared with the average value of all genes (Fig. S4d). The expressions of poly(A)-modifying enzymes were also unchanged in either the transcriptome or translatome (Table S2), suggesting that the translational alterations in the VPA group are not likely attributed to the poly(A) modification.

Gene ontology (GO) analysis revealed that short transcripts (< 1 kb) are highly represented with metabolic genes and long transcripts (≥4 kb) are with synaptic and cytoskeletal genes (Fig. S4e) [49]. Consistently with this notion, VPA-induced upregulated mRNAs, with overall shorter transcript lengths, are enriched with genes related to both mitochondrial proteins, including mitochondrial membrane proteins and those involved in respiration and oxidative phosphorylation, and ribosomal proteins (Fig. 1l, Table S3). On the other hand, VPA-induced downregulated mRNAs, with longer transcript lengths, are enriched with genes related to synaptic membrane and synaptic vesicle (Fig. 1m, Table S3). These findings collectively reveal an imbalanced translational expression profile resulted by in utero VPA exposure, with overrepresentation of mitochondrial and ribosomal genes and shortage of synaptic genes.

### Excessive translation of mitochondrial and ribosomal genes is a common alteration in both VPA-exposed mice and other ASD models

Gene set enrichment analysis (GSEA) further confirmed that gene sets related to ribosome and mitochondria were upregulated, and those related to synapse were downregulated in the cortical translatome of VPA-exposed mice (Fig. 2a, Table S4). To examine whether the mitochondria-, ribosome-, and synapse-related proteins show similar changes as revealed in the translatome, we performed quantitative label-free mass spectrometry on crude synaptosomal proteins from cortical tissues (Fig. S5a). The enrichment of synaptic proteins postsynaptic density protein 95 (PSD95) and Synaptophysin together with a low level of cytosolic protein heat shock protein 90 (HSP90) verified the success of crude synaptosome extraction (Fig. S5b). A total of 521 differentially expressed proteins were identified in the VPA-exposed cortex, and the upregulated proteins were enriched in functional categories related to ATP metabolic processes, mitochondrial protein complex, and ribosome, which is consistent to the altered translatome pattern (Fig. S5c–g). Expression profiles of ribosomal protein, mitochondrial protein, and synaptic assembly gene sets were then compared across the transcriptome, translatome, and proteome by GSEA. Transcriptionally, all three gene sets had no significant alterations by VPA exposure (Fig. 2b–d). However, the ribosomal and mitochondrial proteins were consistently upregulated in both the translatome and proteome of VPA group (Fig. 2b, c). For the synapse assembly gene sets, they were downregulated in the translatome but were upregulated in the proteome of VPA group (Fig. 2d), suggesting more complex mechanisms involved in the regulation of synaptic proteins.

**Fig. 2:**
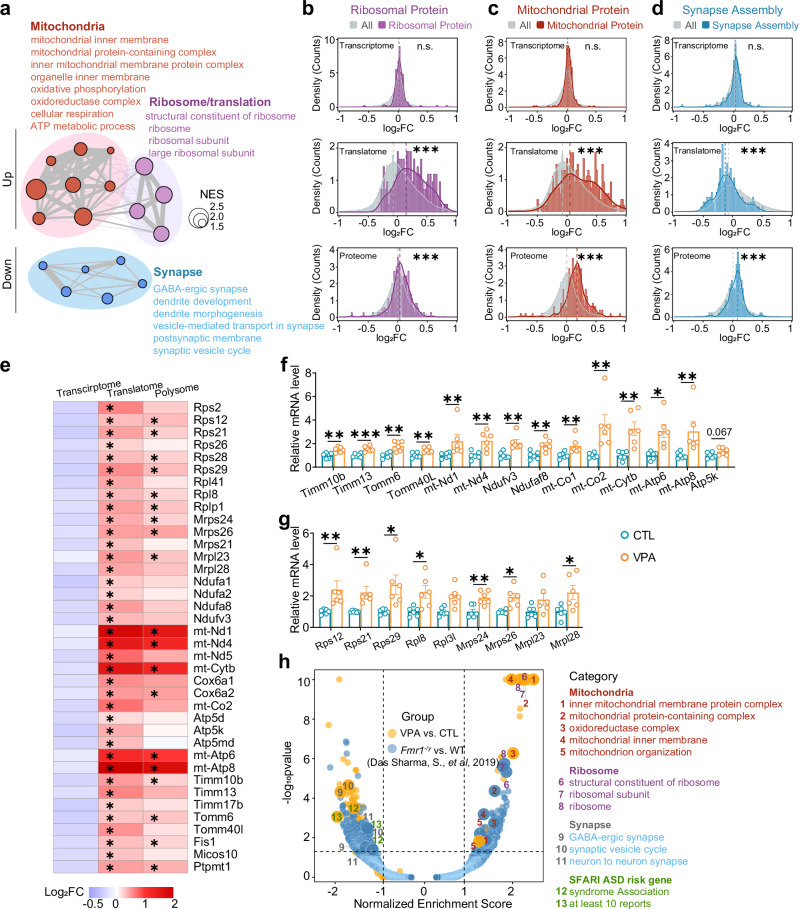
Upregulation of mitochondria- and ribosome-related genes at translational levels in the cerebral cortex of VPA mice. **a** Network diagram of Gene Set Enrichment Analysis (GSEA) showing upregulation of ribosome/translation and mitochondrial-related GO terms, and downregulation of synapse-related GO terms in VPA mice (adjusted *p*-value < 0.05). **b** GSEA comparisons of ribosome gene set (GO:0005840) over the total gene set in VPA vs. CTL datasets. Two-sample z test, in transcriptome, z = 4.0405, not significant (n.s.); in translatome, z = 12.903, ***p < 0.001; in synaptic proteome, z = 3.7872, ***p < 0.001. (**c**) GSEA comparisons of mitochondrial protein-containing complex gene set (GO: 0098798) over the total gene set in VPA vs. CTL datasets. Two-sample z test, in transcriptome, z = 5.9757, not significant (n.s.); in translatome, z = 8.335, ***p < 0.001; in synaptic proteome, z = 12.767, ***p < 0.001. **d** GSEA comparisons of the synapse assembly gene set (GO: 0007416) over the total gene set in VPA vs. CTL datasets. Two-sample z test, in transcriptome, z = 0.99978, not significant (n.s.); in translatome, z = −7.2436, *** *p* < 0.001; in synaptic proteome, z = 3.7049, *** *p* < 0.001. **e** Heatmap showing the Log_2_FC of mRNA expression levels of mitochondria- and ribosome-related genes in the transcriptome, translatome, and the polysome fractions in the cerebral cortex of VPA vs. CTL mice. DEGs are indicated by asterisks. **f,** **g** qPCR verifications of different genes related to mitochondria (**f**) and ribosome (**g**) in the polysome fraction of VPA group. Data are shown as relative mRNA levels of each gene to those of CTL group. CTL, *n* = 6 mice; VPA, *n* = 6 mice. Unpaired *t* test, *p < 0.05, **p < 0.01, ***p < 0.001. Data are expressed as mean ± SEM. **h** Volcano plot showing GSEA of translatome datasets of VPA mice in the current study and *Fmr1*^*−/y*^ mice from Das Sharma, S., et al. [51]. Ribosome/translation- and mitochondria-related GO terms are the most enriched in the upregulated gene sets, and high confident SFARI genes are downregulated. A cutoff value with *p* < 0.05 and |NES| ≥ 1 was used.

We displayed the translational upregulated genes that are related to mitochondria and ribosome in expression heatmaps. Most of these genes also exhibited significantly higher expression in the poly fraction, but all showed no changes at transcriptional levels (Fig. 2e). We further verified the translational levels of these genes by qPCR using the mRNAs isolated from the poly fraction. Indeed, a large proportion of the mitochondria- and ribosome-related mRNAs were notably upregulated in the cortex of VPA group (Fig. 2f, g). To explore whether VPA-induced translational alterations were similarly observed in other ASD models, we performed a joint GSEA analysis of translatome data from both the VPA-exposed mice and two separate studies from the *Fmr1*^*-/y*^ mice [50, 51]. Remarkably, both models share a common upregulation of gene sets involved in mitochondrial function and ribosomal biogenesis (Figs. 2h,  S6a). On the contrary, the high-confidence ASD risk genes listed in the Simons Foundation Autism Research Initiative (SFARI) database, either syndromic or reported in ≥10 independent studies (Table S5 and S6), were significantly downregulated in both the VPA and *Fmr1*^*-/y*^ translatome (Figs. 2h,  S6a). We also compared the proteome of VPA mice with that of Neuroligin 3 (*Nlgn3*) KO mice [28], a widely studied high risk ASD genetic model, and revealed that upregulation of ribosomal proteins were also identified in both models (Fig. S6b). Not only were the translational alterations in VPA model shared with other ASD mouse models, they were also observed in human samples. Notably, the upregulated translation in mitochondrial energy metabolism pathways was overlapped with the translatome results from both the patient brain samples and patient-derived neural progenitor cells of TSC [52] (Fig. S6c). These cross-model and cross-species analyses together demonstrate that enhanced translation and overproduction of ribosomal and/or mitochondrial components constitute shared molecular hallmarks across diverse ASD etiologies from environmental (VPA) and monogenic (*Fmr1*, *Nlgn3, or TSC1/2*) origins.

### VPA mice exhibit altered mitochondrial and synaptic morphology in the cerebral cortex

The upregulation of mitochondrial protein complexes and oxidative phosphorylation in both the translatome and synaptic proteome of VPA-exposed cortex prompted us to further examine the potential morphological and functional changes of mitochondria in the VPA group. Measurements of ATP level, serving as a direct indicator of cellular energy metabolism, revealed an overall increase of ATP production in the cortex of the VPA mice (Fig. 3a). To determine the morphological changes of mitochondria, we focused on the medial prefrontal cortex (mPFC), a pivotal subregion of the frontal cortex intricately involved in social behaviors and highly vulnerable in ASD [53]. Transmission electron microscopy (TEM) was utilized to examine the ultrastructure of mitochondria in the synaptic area of mPFC in VPA mice. Compared to the control group, a notable increase of mitochondrial area, the aspect ratio, and the number of mitochondria per unit area within synaptic regions was observed in the mPFC of VPA mice (Fig. 3b–f). Furthermore, a significant increase of presynaptic area and presynaptic diameter along with fewer synaptic vesicles per unit area was observed in the asymmetric synapses of VPA group compared to the control group (Fig. 3g–l). A trend towards increased synaptic length was also observed, whereas perimeters in the postsynaptic sites were not changed significantly (Fig. 3m–o). These results collectively suggest morphological changes in synaptic mitochondria and presynaptic structures of the mPFC in VPA mice, which was consistent with the findings that mitochondria and presynaptic gene changes were enriched in the translational DEGs of VPA group.

**Fig. 3:**
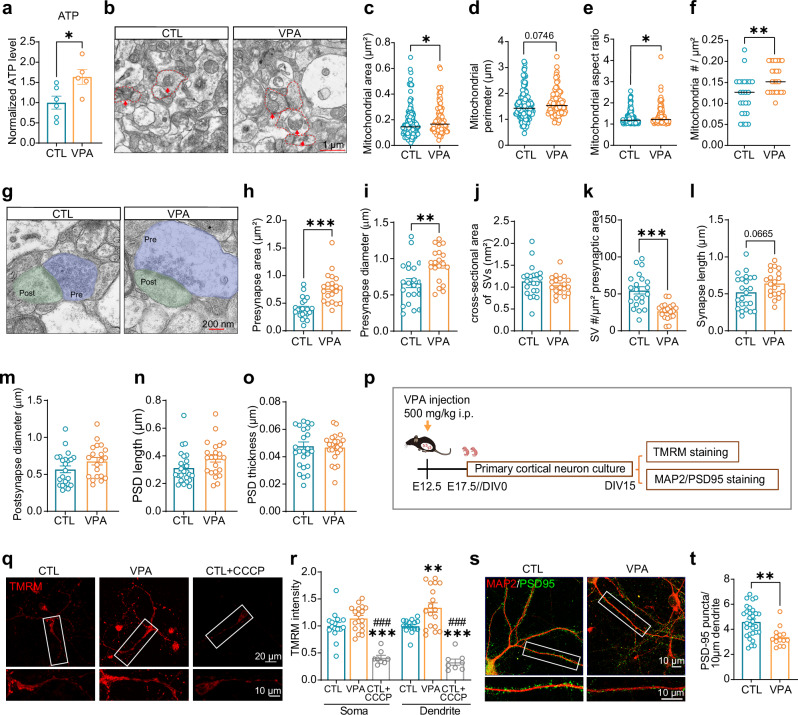
VPA mice exhibit altered mitochondrial and synaptic morphology in the cerebral cortex. **a** Normalized ATP levels in the cerebral cortex of CTL and VPA mice at P35. CTL, *n* = 6 mice; VPA, *n* = 5 mice. Unpaired *t* test, *p < 0.05. **b** Representative transmission electron microscope (TEM) micrograph of the medial prefrontal cortex (mPFC) of CTL and VPA mice at P35. Synapse areas are framed by red dotted lines. Mitochondria are marked by red arrows. Scale bar,1 μm. **c**–**f** Quantification analysis of mitochondrial area (**c**), mitochondrial perimeter (**d**), mitochondrial aspect ratio (**e**), and mitochondrial density (**f**) in synapse regions in the mPFC of 5-week-old CTL and VPA mice. *n* = 3 mice in each group, and at least 10 images were taken for analysis per mouse. Unpaired *t* test, *p < 0.05, **p < 0.01. **g** Representative TEM micrographs of the synapse regions in the mPFC. Presynapse areas are highlighted in purple and postsynapse areas green. Scale bar, 200 nm. (**h**–**o**) Quantification analysis of presynapse area (**h**), presynapse diameter (**i**), cross-sectional area of synaptic vesicles (SVs; **j**), number (No.) of SVs/unit area (**k**), synapse length (**l**), postsynapse diameter (**m**), postsynaptic density (PSD) length (**n**), and PSD thickness (**o**) in the mPFC of 5-week-old CTL and VPA mice. *n* = 3 mice in each group, and at least 10 images were taken for analysis per mouse. Unpaired *t* test, **p < 0.01, ***p < 0.001. **p** Schematic illustration of experiments in primary cortical neuron culture. **q** Representative image of TMRM (tetramethylrhodamine) staining at 14 DIV of cultured primary cortical neurons. CCCP (carbonyl cyanide m-chlorophenyl hydrazone), an agent causing loss of mitochondrial membrane potential, acts as the negative control. Scale bars: upper panels, 20 μm; lower panels (magnified from the rectangle areas in the upper panels), 10 μm. **r** Quantification analysis of TMRM signal intensities from three independent experiments. Kruskal-Wallis test for soma area, ***p < 0.001, CTL vs. CTL + CCCP group; ### *p* < 0.001, VPA vs. CTL + CCCP group. Brown-Forsythe and Welch ANOVA for dendrite area, **p < 0.01, ***p < 0.001, VPA group or CTL + CCCP vs. CTL; ### *p* < 0.001, CTL + CCCP vs. VPA. **s** Representative images of MAP2 (microtubule associated protein 2; a dendrite marker) and PSD95 (postsynaptic density protein 95, a postsynaptic marker) staining at 14 DIV of cultured primary cortical neurons. Scale bars, 10 μm. **t** Quantification analysis of PSD95 puncta density from three independent experiments. Unpaired *t* test, **p < 0.01. Data are expressed as mean ± SEM.

We further explored whether the observed mitochondrial and synaptic alterations occurred in cortical neurons by in utero exposure of VPA. VPA (500 mg/kg) was similarly i.p. administered to pregnant mice on E12.5, and primary cortical neurons were prepared from the entire cortex on E17.5 and cultured for 15 days (Fig. 3p). Increased fluorescent intensity of tetramethylrhodamine (TMRM), a mitochondrial membrane potential indicator, was observed in the dendrites of VPA-modeled neurons when compared to the control group, whereas no difference of TMRM signals was found in the soma (Fig. 3q, r). Furthermore, the synaptic density, labeled by the postsynaptic marker PSD95, was substantially decreased in the VPA-modeled neurons (Fig. 3s, t). These findings confirmed elevated dendritic mitochondrial function and reduced synaptic numbers in cortical neurons following in utero VPA treatment.

### Activation of mTOR-p70S6K-RPS6 and MNK-eIF4E pathways in the cortex of VPA-exposed offspring

Given that protein synthesis is upregulated by in utero VPA exposure, we sought to explore whether mRNA translational regulatory pathways are altered in the VPA group. The major translational regulatory signals are conveyed by mTORC1, which leads to a phosphorylation signaling cascade including p70 ribosomal protein S6 kinase (p70S6K) and ribosomal protein S6 (RPS6), known to promote ribosome biogenesis. mTORC1 also induces phosphorylation of eIF4E-binding protein (4EBP), a repressor of eIF4E, hence relieving the inhibitory effect toward eIF4E and allowing cap-dependent translation initiation. Additionally, eIF4E is activated by the extracellular signal-regulated kinase (ERK)-MNK signaling cascade within the mitogen-activated protein kinases (MAPK) pathway. Phosphorylation of eIF4E at Ser209 by MNK is an important positive regulatory site for protein synthesis. Because dysregulation of these signaling pathways is tightly associated with ASD pathology, we investigated them in the cerebral cortex of VPA-exposed offspring at P35 (Figs. 4a–i, S7a–g). First, the mTORC1-p70S6K-RPS6 signaling axis was activated shown by the upregulated phosphorylation of mTOR, p70S6K, and RPS6 in the VPA group (Figs. 4a–d, S7a–c), which was consistent with several previous reports [40–43]. Additionally, although the phosphorylation of 4EBP1 was not altered (Figs. 4a, S7d), the total expression of the 4EBP1/2 was significantly decreased (Fig. 4a, e, f), suggesting activation of eIF4E. Indeed, the positive regulatory S209 phosphorylation of eIF4E was prominently increased in the VPA group (Figs. 4a, g, S7e). Phosphorylation of ERK and MNK was not significantly changed, but the total protein level of MNK showed an increasing trend (Figs. 4a, h, i, S7f, g). Therefore, the decrease of 4EBP1 and increase of MNK, together with the elevated eIF4E phosphorylation, collectively suggest an activation of eIF4E-dependent translation initiation in the cortex of VPA-exposed mice offspring. To determine when this activation emerges during development and whether it occurs in both sexes, we tracked p-eIF4E levels in male and female cortices at E18.5, P12, and P45. Remarkably, a significant increase of Ser209 phosphorylation of eIF4E was detected only in male VPA mice at P45 (Fig. S8a–i).

**Fig. 4:**
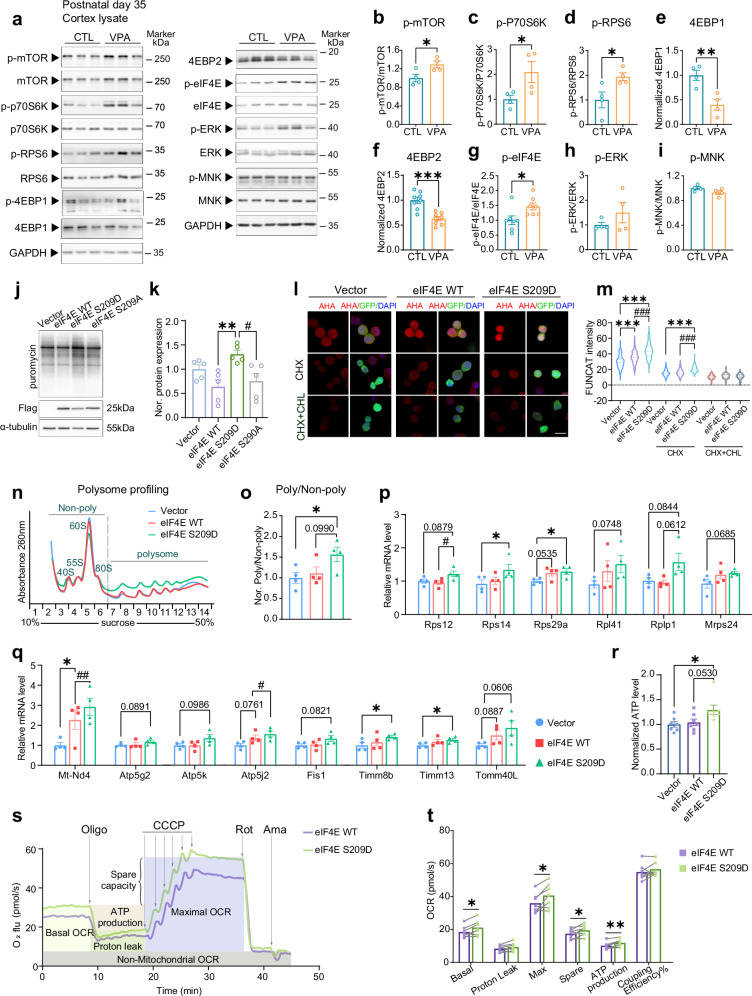
eIF4E activation leads to increased ribosomal/mitochondrial protein synthesis and enhanced mitochondrial respiration. **a** Representative immunoblots of proteins of mTOR-p70S6K-RPS6, mTOR-4EBP1/2-eIF4E, and ERK-MNK-eIF4E pathways in the cerebral cortex of CTL and VPA mice at P35. GAPDH was used as a loading control. **b**–**i** Quantification of protein expressions and phosphorylation levels of different molecules of MNK-eIF4E and mTOR-p70S6K-RPS6 pathways. Unpaired *t* test, *p < 0.05, **p < 0.01. **j,** **k** Representative immunoblots (**j**) and quantification analysis (**k**) of puromycin-labeled de novo global protein synthesis in Neuro-2a cells transfected with Vector, eIF4E-WT, eIF4E-S209A, and eIF4E-S209D from four independent experiments. α-tubulin was used as a loading control. One-way ANOVA, *p < 0.05, eIF4E-S209D vs. Vector; ##*p* < 0.01, eIF4E-S209D vs. eIF4E-WT; & &*p* < 0.01, eIF4E-S209D vs. eIF4E-S209A. **l,** **m** Representative images (**l**) and signal intensity quantification analysis (**m**) of L-azidohomoalanine (AHA) labeling of newly synthesized proteins treated with cycloheximide (CHX) and with or without chloramphenicol (CHL) in Neuro-2a cells, transfected with Vector, eIF4E-WT, and eIF4E-S209D from four independent experiments. At least 30 cells/group were analyzed in each experiment. One-way ANOVA, ***p < 0.001, eIF4E-WT or eIF4E-S209D vs. Vector; ### *p* < 0.001, eIF4E-S209D vs. eIF4E-WT. Scale bar, 20 μm. (**n,** **o**) Representative polysome profiling (**n**) and quantification of polysome/ (60S + 80S) ratio (**o**) from Neuro-2a cells transfected with Vector, eIF4E-WT, and eIF4E-S209D from four independent experiments. **p,** **q** qPCR verification of different genes related to ribosome and mitochondrial in the polysome fraction from Neuro-2a cells transfected with Vector, eIF4E-WT, and eIF4E-S209D from four independent experiments. Data are shown as relative mRNA levels of each gene to those of Vector group. Unpaired *t* test, *p < 0.05, eIF4E-WT or eIF4E-S209D vs. Vector; # *p* < 0.05, ## *p* < 0.01, eIF4E-S209D vs. eIF4E-WT. **r** The normalized ATP levels in Neuro-2a cells transfected with Vector, eIF4E-WT, and eIF4E-S209D from three independent experiments. One-way ANOVA, *p < 0.05. **s,** **t** Representative traces (**s**) and quantification of oxygen consumption rate (OCR; **t**) from HEK293T cells transfected with eIF4E-WT and eIF4E-S209D from four independent experiments. Unpaired *t* test, *p < 0.05, **p < 0.01. Data are expressed as mean ± SEM.

### eIF4E activation recapitulates increased ribosomal/mitochondrial protein synthesis and enhanced mitochondrial respiration

To understand whether eIF4E activation leads to altered mitochondrial protein synthesis and function, we overexpressed constructs encoding the wild-type (WT) form, a non-phosphorylatable (Ser209 mutated to Ala, or S209A) mutant, and a phosphomimetic (Ser209 mutated to Asp, or S209D) mutant of eIF4E in Neuro-2a cells. The total protein synthesis levels, shown by the puromycylated nascent peptide amount, were not significantly changed in eIF4E-WT or S209A-expressing cells, but was prominently promoted by eIF4E-S209D (Fig. 4j, k). To visualize cytosolic and mitochondrial protein synthesis, we took advantage of the fluorescent non-canonical amino acid tagging (FUNCAT) method. AHA was added to methionine-starved cells to label de novo proteins in both the cytosol and mitochondria, and then clicked with a 546 fluorophore. eIF4E-WT and S209D both increased the total protein synthesis, and the effect of S209D was stronger than WT (Fig. 4l, m). Mitochondria-specific translation was revealed by the remaining FUNCAT signals after selective inhibition of cytosolic translation with cycloheximide, a specific cytosolic eukaryotic translation inhibitor. Only eIF4E-S209D-expressing cells showed increased mitochondrial protein synthesis (Fig. 4l, m). FUNCAT signals were nearly absent when both cycloheximide and the mitochondrial translation inhibitor chloramphenicol were added to the cells, indicating the authenticity of the signals for labeling de novo protein synthesis (Fig. 4l, m). These data suggest that eIF4E activation leads to increased cytosolic and mitochondrial translation. Polysome profiling also showed an increased ratio of poly over non-poly peak areas in S209D-expressing cells relative to vector- and WT-expressing cells (Fig. 4n, o). To verify the expression levels of genes associated with mitochondria and ribosomes in eIF4E activation group, mRNA was isolated from the translationally active poly fraction. Subsequent qPCR analysis confirmed that mRNA levels of the ribosome- (Fig. 4p) and mitochondria-related genes (Fig. 4q) were both translationally upregulated in S209D-expressing cells.

Having validated that eIF4E-S209D promoted mitochondrial mRNA translation, we went on to examine the mitochondrial function. Cells expressed with eIF4E-S209D exhibited an increased production of ATP compared to those with vector or eIF4E-WT (Fig. 4r). Mitochondrial respiration was then evaluated as oxygen consumption rate (OCR) using Oroboros O2k in HEK293T cells transfected with eIF4E-WT or eIF4E-S209D. Indeed, compared to eIF4E-WT, eIF4E-S209D promoted the basal respiration rate, along with increased maximal OCR, spare respiratory capacity, and ATP production (Fig. 4s, t). Taken together, overexpression of the active eIF4E form, eIF4E-S209D, led to increased global protein synthesis, upregulated translation of ribosomal and mitochondrial genes, and elevated cellular mitochondria respiration with more ATP production.

### Correction of eIF4E hyperphosphorylation during juvenile has long-lasting effect in reversing ASD-like behaviors in VPA-exposed offspring

Because the above-described observations in eIF4E-S209D-expressing cells recapitulated the translational dysregulation in the cortex of VPA-exposed offspring, we investigated whether correction of eIF4E hyperphosphorylation could rescue the abnormalities in the animals. eFT508, a blood-brain barrier penetrable inhibitor of MNK1/2, has been shown to rescue ASD-like behavior deficits in *Fmr1* KO and *synaptic Ras GTPase activating protein 1* (*Syngap1)*^+/−^ mice [29, 54]. We first confirmed that i.p. injection of eFT508 (1 and 2 mg/kg) for 1 h could potently inhibit Ser209 phosphorylation of eIF4E in the cortex of juvenile mice (P25; Fig. S9a–c). The spontaneous exploratory locomotor activity of mice in the Open field test was unaffected after eFT508 treatment (Fig. S9d, e), suggesting no significant side effects on the general behaviors of mice.

Before drug administration, we subjected the weaned VPA-exposed offspring (3 weeks old) to a single run of social discrimination test to confirm success of the modeling (Fig. S9f, g). We then administered a continuous 4-day i.p. injections of eFT508 at a dose of 1 mg/kg (or vehicle as a control) at 3–4 weeks of age, followed by a series of behavioral tests conducted at 4–5 weeks. An additional dose of eFT508 was administered 1 h before each test (Fig. 5a). Post-hoc analysis at 5 weeks old confirmed that this juvenile treatment paradigm effectively reversed the upregulated cortical Ser209 phosphorylation of eIF4E in the VPA group both by Western blotting and immunofluorescence (Fig. 5b–e). Importantly, eFT508 treatment substantially restored both the social approach and social novelty score in VPA-expose mice in the Three-chamber test (Fig. 5f, g). The stereotyped self-grooming of VPA mice was also significantly reduced by the treatment of eFT508 (Fig. 5h). However, eFT508 did not improve the anxiety-like behaviors in VPA mice in the elevated Zero-maze test (Fig. S9h, i). eFT508 also did not affect the exploratory locomotor activity of the mice in the Open field test (Fig. S9j, k). To test whether this juvenile treatment regime of eFT508 has long-lasting effect, we waited until the mice reached adulthood (9 weeks old) after the initial behavior tests, and retested them for Three-chamber social interaction and self-grooming. Notably, juvenile treatment of eFT508 persistently exhibited potent effects on reversing the elevated cortical Ser209 phosphorylation of eIF4E in the VPA group at adult (Fig. 5i, j). More importantly, the rescue effects of the drug on social deficits and stereotyped behavior were both sustained until adulthood, indicated by the reversed social approach and self-grooming time in VPA mice at 9 weeks old (Fig. 5k–m). Collectively, eFT508 treatment at juvenile exhibits both short-term and long-lasting efficacy on restoring normal social behaviors and reducing stereotyped behavior in VPA-exposed offspring mice.

**Fig. 5:**
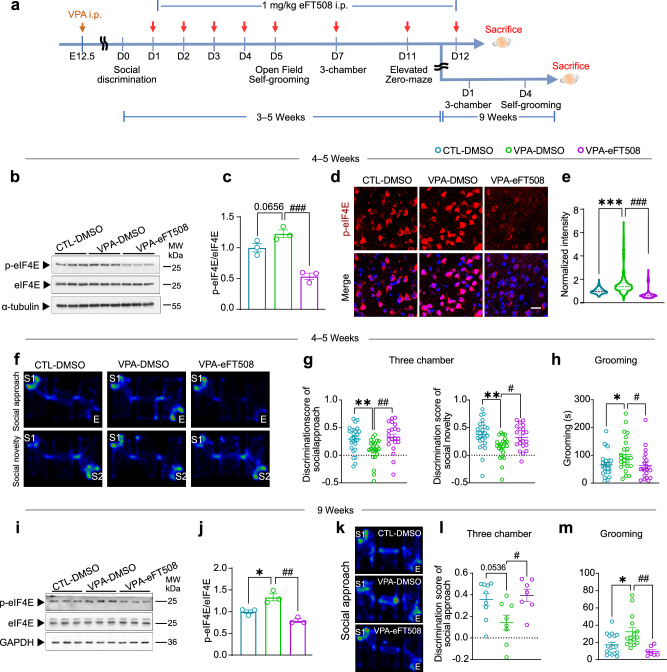
Administration of eFT508 during juvenile reverses social deficits and repetitive stereotyped behaviors in VPA mice. **a** Timeline of experimental procedures of drug treatment and different analyses. **b,** **c** Representative immunoblots (**b**) and qualification (**c**) of p-eIF4E (Ser209) levels in the cerebral cortex of CTL-DMSO, VPA-DMSO, and VPA-eFT508 mice at 5 weeks old. α-tubulin was used as a loading control. *n* = 3 mice in each group. One-way ANOVA, *p* = 0.0656, CTL-DMSO vs. VPA-DMSO; ###*p* < 0.001, VPA-eFT508 vs. VPA-DMSO. **d,** **e** Representative images (**d**) and intensity quantification analysis (**e**) of p-eIF4E in the cerebral cortex of 6-weeks-old mice. CTL-DMSO, *n* = 5 mice; VPA-DMSO, *n* = 4 mice; VPA-eFT508, *n* = 4 mice. Two brain slices per mouse, and 2–4 fields of view per slice. One-way ANOVA, ***p < 0.001, CTL-DMSO vs. VPA-DMSO; ###*p* < 0.001, VPA-eFT508 vs. VPA-DMSO. Scale bar, 20 μm. **f** Representative activity heatmaps of CTL-DMSO, VPA-DMSO, and VPA- eFT508 mice in social approach (upper panels) and social novelty (lower panels) stage of Three-chamber test at 4–5 weeks old. E: empty cup; S1: stranger #1 contained cup; S2: stranger #2 contained cup. **g** Discrimination score of social approach (left panel), calculated by the difference of time spent in sniffing S1 and E, and discrimination score of social novelty preference (right panel), calculated by the difference of time spent in sniffing S2 and S1, of CTL-DMSO, VPA-DMSO, and VPA- eFT508 mice at 4–5 weeks old. CTL-DMSO, *n* = 26 mice; VPA-DMSO, *n* = 22 mice; VPA-eFT508, *n* = 21 mice. Kruskal-Wallis test. **p < 0.01, VPA-DMSO vs. CTL-DMSO; #*p* < 0.05, ##*p* < 0.01, VPA-eFT508 vs. VPA-DMSO. **h** Duration of self-grooming of CTL, VPA, and VPA-eFT508 mice at 4–5 weeks old. CTL-DMSO, *n* = 28 mice; VPA-DMSO, *n* = 24 mice; VPA-eFT508, *n* = 21 mice. Kruskal-Wallis test. #*p* < 0.05, VPA-eFT508 vs. VPA. **i,** **j** Representative immunoblots (**i**) and qualification (**j**) of p-eIF4E levels in the cerebral cortex of CTL-DMSO, VPA-DMSO, and VPA-eFT508 mice at 9 weeks old. GAPDH was used as a loading control. *n* = 3 mice in each group. One-way ANOVA, *p < 0.05, CTL-DMSO vs. VPA-DMSO; ##*p* < 0.01, VPA-eFT508 vs. VPA-DMSO. **k,** **l** Representative activity heatmaps (**k**) and discrimination score of social approach (**l**) of CTL-DMSO, VPA-DMSO, and VPA-eFT508 mice in social approach stage of Three-chamber test at 9 weeks old. CTL-DMSO, *n* = 9 mice; VPA-DMSO, *n* = 8 mice; VPA-eFT508, *n* = 7 mice. One-way ANOVA, *p* = 0.0536, VPA-DMSO vs. CTL-DMSO; #*p* < 0.05, VPA-eFT508 vs. VPA-DMSO. **m** Duration of self-grooming of CTL-DMSO, VPA-DMSO, and VPA-eFT508 mice at 9 weeks old. CTL-DMSO, *n* = 17 mice; VPA-DMSO, *n* = 17 mice; VPA-eFT508, *n* = 7 mice. Kruskal-Wallis test. *p < 0.05, VPA-DMSO vs. CTL-DMSO; ##p < 0.01, VPA-eFT508 vs. VPA-DMSO. Data are expressed as mean ± SEM.

### Correction of eIF4E hyperphosphorylation reverses excessive translation of mitochondrial and ribosomal mRNAs in VPA-exposed offspring

Following eFT508 treatment and behavior analysis during juvenile, we quantified puromycin-labeled nascent polypeptide levels in cortical tissues across experimental groups. While the VPA-DMSO group exhibited elevated de novo protein synthesis compared to the Control-DMSO group, the VPA-eFT508 group displayed partially restored cortical protein synthesis levels (Fig. 6a, b). Immunofluorescent analysis corroborated that the puromycin incorporation was notably augmented in the cortex of VPA-DMSO group, but reduced post-treatment of eFT508 (Fig. 6c, d). To elucidate the impact of eFT508 on mRNA translational profile in the cerebral cortex of VPA mice, polysome profiling was employed for translatome analysis. Peak area measurements consistently revealed an increased ratio of poly over non-poly fractions in VPA mice, and eFT508 treatment restored the area ratio to normal level (Fig. 6e). Translatome analysis by polysome profiling identified 948 DEGs in VPA-eFT508 group relative to VPA-DMSO group, including 382 upregulated and 566 downregulated genes (Fig. S10a, Table S7). The parallel bulk RNA-seq revealed only 25 DEGs at transcription level, showing almost no overlap with the translational DEGs, in agreement with the notion that eIF4E is a translational regulator (Fig. 6f, Fig. S10b). Notably, eFT508 treatment led to length-dependent translational changes which is opposite to those induced by VPA, that is, shorter transcripts were more downregulated by eFT508 treatment, indicated by the significant left-shift of the cumulative distribution curve of CDS < 1 kb transcripts than that of CDS ≥ 4 kb transcripts (Fig. 6g). Consistently, the downregulated DEGs were enriched with gene sets associated with mitochondrial proteins and ribosomes (Fig. S10c, d). Subsequently, we performed a joint analysis of the VPA vs. control DEGs and eFT508 vs. vehicle DEGs, and identified 183 reversed DEGs in which 166 were upregulated by VPA but downregulated by eFT508 (Fig. 6h, Table S8). GO analysis and GSEA of these reversed DEGs showed enrichment of mitochondrial and ribosomal genes (Fig. 6i, j, Table S9). The polysome mRNA expression heatmaps revealed individual mitochondrial and ribosomal genes that were translationally upregulated by VPA but reversed by eFT508 (Fig. 6k), and most of them were validated by qPCR analysis (Fig. 6l, m).

**Fig. 6:**
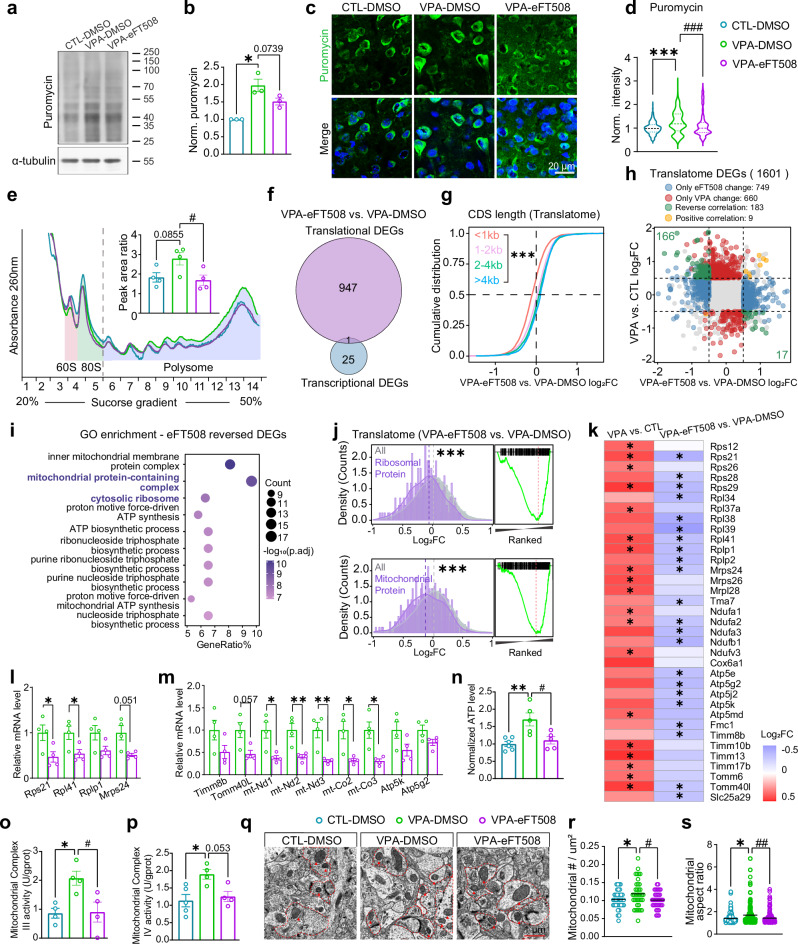
Administration of eFT508 reverses exaggerated protein synthesis in VPA Mice. **a,** **b** Representative immunoblots (**a**) and qualification (**b**) of puromycin-labeled de novo global protein synthesis in cerebral cortical slices of mice at 6 weeks old. α-tubulin was used as a loading control. *n* = 3 mice in each group. One-way ANOVA, *p < 0.05, VPA-DMSO vs. CTL-DMSO; *p* = 0.0739, VPA-eFT508 vs. VPA-DMSO. **c,** **d** Representative images (**c**) and intensity quantification (**d**) of puromycin in the cerebral cortex of CTL-DMSO, VPA-DMSO, and VPA-eFT508 mice at 6 weeks old. Two brain slices were stained from each mouse, and 2–4 fields of view within the somatosensory regions were photographed from each slice. CTL-DMSO, *n* = 5 mice; VPA-DMSO, *n* = 4 mice; VPA-eFT508, *n* = 4 mice. One-way ANOVA, ***p < 0.001, CTL-DMSO vs. VPA-DMSO; ###*p* < 0.001, VPA-eFT508 vs. VPA-DMSO. Scale bar, 20 μm. **e** Representative polysome profiling chart and quantification of polysome/(60S + 80S) ratio from the cerebral cortex of mice at 9 weeks old. *n* = 4 mice in each group. One-way ANOVA, *p* = 0.0855, VPA-DMSO vs. CTL-DMSO; #*p* < 0.05, VPA-eFT508 vs. VPA-DMSO. **f** Venn diagram showing little overlap of transcriptional and translational DEGs. **g** The Log_2_FC (VPA-eFT508 vs. VPA-DMSO) of different CDS length populations in the translatome are shown as cumulative distribution curves. Two-sided KS test, < 1 kb vs. ≥ 4 kb, D = 0.2917, ***p < 0.001. **h** Log_2_FC of translational DEGs of VPA vs. CTL groups and DEGs of VPA-eFT508 vs. VPA-DMSO groups. **i** Bubble plot showing GO enrichment of translational DEGs that are changed in VPA group but reversed by eFT508 treatment. **j** GSEA comparisons of the ribosome (GO:0005840, NES = 2.153786, FDR = 1.765 × 10^−8^) and mitochondrial protein-containing complex gene sets (GO:0098798, NES = −1.704, FDR = 0.002) over the total gene sets (VPA-eFT508 vs. VPA-DMSO). Two-sample z test, z = −5.9672, ***p < 0.001, ribosome; z = −5.2805, ***p < 0.001, mitochondrial protein-containing complex. **k** Heatmap showing upregulated genes in VPA vs. CTL groups, but downregulated in VPA-eFT508 vs. VPA-DMSO groups in the polysome fractions of the cerebral cortex. DEGs are indicated by asterisks. **l,** **m** qPCR verification of upregulation of ribosome- (**l**) and mitochondria- (**m**) related genes in the polysome fraction of VPA-eFT508 group. Data are shown as relative mRNA level of each gene to that of VPA-DMSO group. *n* = 4 mice in each group. Unpaired *t* test, *p < 0.05, **p < 0.01. **n** The normalized ATP levels in the cerebral cortex of 9-week-old mice of different groups. *n* = 3 mice in each group. One-way ANOVA, **p < 0.01, VPA-DMSO vs. CTL-DMSO; #*p* < 0.05, VPA-eFT508 vs. VPA-DMSO. **o,** **p** Mitochondrial Complex III (**o**) and IV (**p**) activity in the cerebral cortex of 6-week-old mice of different groups. *n* = 4 mice in each group. One-way ANOVA, *p < 0.05, VPA-DMSO vs. CTL-DMSO; *p* = 0.053, #*p* < 0.05, VPA-eFT508 vs. VPA-DMSO. **q**–**s** Representative TEM micrographs (**q**) and quantification analysis of mitochondrial aspect ratio (**r**) and mitochondrial density (**s**) in the synapse region of mPFC of 5-week-old VPA-DMSO and VPA-eFT508 mice. Scale bar, 200 nm. *n* = 4 mice in each group, and at least 10 images were analyzed per mouse. Unpaired *t* test, *p < 0.05, VPA-DMSO vs. CTL-DMSO; #*p* < 0.05, ##*p* < 0.01, VPA- eFT508 vs. VPA-DMSO. Data are expressed as mean ± SEM.

We further assessed the effects of eFT508 on mitochondrial function and morphology in the cerebral cortex of VPA mice. Importantly, eFT508 corrected the ATP overproduction in the VPA mice (Fig. 6n). Moreover, assessment of the mitochondrial respiratory chain complex activities showed a significant elevation in the activities of Complex III and Complex IV in the VPA cortex compared with the control group, which were normalized following eFT508 administration (Fig. 6o, p). The activities of the remaining complexes did not exhibit significant changes (Fig. S11a–c). Finally, TEM analysis revealed that the mitochondria number and mitochondrial aspect ratio in the mPFC synaptic compartment of VPA mice were both reversed by eFT508 (Fig. 6q–s). Taken together, these results confirmed that the juvenile eFT508 treatment acts effectively on the translatome and normalizes the translation of mitochondrial/ribosomal genes and mitochondrial function in the cortex of VPA mice.

## Discussion

This study provides the first characterization of the pathophysiology of protein synthesis perturbation in VPA-induced ASD offspring model. Using multiple protein synthesis measurement approaches, we demonstrate an exaggerated overall de novo protein synthesis in the cerebral cortex of VPA-induced offspring during juvenile stages. We further revealed translatome changes in the VPA cortex by integrative analysis of data obtained from polysome profiling. An imbalanced alteration of translatome, but not transcriptome, was identified in the VPA cortex, characterized by the excessive translation of short transcripts overrepresented by ribosomal and mitochondrial genes, and downregulation of long transcripts enriched with synaptic genes. Further analysis pinpoints eIF4E, which is overactivated in the VPA-exposed cortex, as a key factor to induce translational upregulation of ribosomal and mitochondrial genes, mitochondria impairments, and core ASD-like behaviors in VPA mice.

The imbalanced disruption of translatome in the VPA-exposed offspring was supported by observations in other genetic ASD models and ASD human subjects (Table 1). A highly similar translatome pattern was reported in the hippocampal CA1 neurons in *Fmr1*^*-/*y^ mice, with short metabolic mRNAs highly translated, including ribosomal and mitochondrial mRNAs, and long synaptic mRNAs translationally downregulated [49]. Additionally, excessive ribosome proteins were produced in the ventral tegmental area of mice with knockout of the ASD high-risk gene *Nlgn3* [28]. Clinical evidence has revealed that ASD-related functional connectivity is linked to regional differences in the expression of gene sets related to translation process and ribosomal proteins [55]. Moreover, upregulation of ribosomal genes is consensually found in studies using post-mortem cortical tissues and induced pluripotent stem cell-derived neural progenitor cells from ASD subjects [56–58]. Additionally, increase of ribosomal gene modules is closely associated with downregulated synaptic-enriched gene modules in the cortical transcriptome of ASD subjects [59]. The overproduction of ribosome proteins is also hypothesized with enhanced ribosomal biogenesis contributed from higher gene dosage of ribosomal RNA (rRNA) in ASD [60]. The current study provides further evidence from a classic environmental ASD model, reinforcing that upregulation of ribosomal proteins is a consensus pathological feature of ASD.

**Table 1:** Comparisons of the key pathological changes in this study and others.

VPA-induced mice offspring (this study)	ASD individuals or animal models	Species	Ref.
Exaggeration of protein synthesis and overproduction of ribosomal proteins in the cerebral cortex	Upregulation of ribosomal genes in postmortem cortical tissues and induced pluripotent stem cell-derived neural progenitor cells from ASD subjects	Human	[56–59]
	Excessive translation of ribosomal genes in the cortex and hippocampus of *Fmr1* KO mice	Mouse	[49–51]
	Overproduction of ribosomal proteins in ventral tegmental area of *Nlgn3* KO mice	Mouse	[28]
	Upregulation of protein sets related to translation, ribosome, and rRNA synthesis in the embryo proper following 4–8 h exposure of VPA	Mouse	[61]
Upregulated translation of mitochondrial genes and increased mitochondrial function in the cerebral cortex	Increased metabolic and mitochondrial gene expressions in TSC patient brain samples and derived cells	Human	[52]
	Excessive translation of mitochondrial genes in the cortex and hippocampus of *Fmr1* KO mice	Mouse	[49–51]
	Elevated abundance of mitochondrial ribosomal proteins in *Nlgn3* KO mice	Mouse	[28]
	Increased respiratory capacity and mitochondrial mass in the PFC neurons of MIA-induced offspring	Mouse	[70]
	Elevated mitochondrial activity in mutants of the human *CYFIP1* homolog	Drosophila	[86]
	Enhanced mitochondrial RNA metabolic process in male *Adnp*^+/-^ mice (Data reanalyzed and shown in Fig. S12)	Mouse	[71]
	Increased mitochondrial respiration in neurons deficient with ASD risk genes *Taok2*, *Syngap1*, or *Etfb*	Mouse	[74]
Exaggerated eIF4E phosphorylation (S209) in the cerebral cortex, and inhibition of eIF4E phosphorylation reversed translatome abnormalities and ASD-like behaviors	Enhanced *EIF4E* promoter activity in ASD, and eIF4E phosphorylation levels positively correlate with the severity of ASD	Human	[12–15]
	Increased eIF4E phosphorylation in TSC patient-derived cells	Human	[26]
	eIF4E phosphorylation elevated in postmortem brains from FXS patients and *Fmr1* KO mice	Human, Mouse	[22, 23, 25]
	eIF4E mRNA increased in male *ANDP*+/- mice	Mouse	[24]
	eIF4E phosphorylation elevated in MIA offspring	Mouse	[25]
	eIF4E overexpression or hyperphosphorylation directly drives ASD-like behaviors	Mouse	[7, 8, 17–19]
	Pharmacological or genetic downregulation of eIF4E reversed ASD-like behaviors	Mouse	[22, 27–30]

As a histone deacetylase (HDAC) inhibitor, VPA is capable of rapidly modulating gene expression at the epigenetic level. A study reported that 4–8 h exposure of VPA to whole mouse embryos culture leads to significant upregulation of protein sets related to translation, ribosome, and rRNA synthesis in the embryo proper [61]. On the contrary, it has been found that direct addition of VPA to cell cultures for 24 h, such as human neural progenitor cells and the SH-SY5Y neuroblastoma cells, leads to downregulation of ribosomal gene set [62, 63]. This discrepancy may originate from the different responses to VPA between an organ-wide system and a single cell population. Given this consideration, this study employed the same VPA administration route as used in the animal model prior to neuronal culture preparations. We propose that this cell modeling approach may yield results more closely resembling in vivo pathologies. Another potential reason for the inconsistent findings might be the different time windows of the investigations. In a model of MIA, another high-risk environmental factor of ASD, 4 h post-injection of lipopolysaccharide (LPS) at E15 leads to remarkable increase of translation and cell cycle-related genes whereas decrease of SFARI ASD genes in rat fetal brain. However, very little DEGs were found at 24 h post-injection of LPS [64]. These findings suggest that although prenatal insults may induce transient alterations in the developing fetal brain, they could lead to long-term damage in the offspring due to the critical neural proliferation stages involved.

Since clinical symptoms in ASD individuals typically manifest during infancy to juvenile stages, investigating gene expression changes within this specific time window may greatly aid in pinpointing the causative factors underlying the symptoms. In the current study, we revealed that alterations of both eIF4E hyperphosphorylation and global translation emerge at the transition from juvenile to adolescence (P12 to P35/P45). Importantly, the exaggerated translation of short metabolic genes as well as the mitochondrial dysfunction in juvenile VPA-exposed offspring provide novel evidence for the pathology of ASD. It is worth noting that the translationally upregulated transcripts in the VPA group not only possess shorter CDS, but also shorter 5’UTR and 3’UTR. It has been shown that shorter 5’UTRs lack complex RNA secondary structures, thus facilitating faster scanning of the translation initiation complex for the start codon, thereby enhancing translation initiation efficiency [65, 66]. Similarly, shorter 3’UTRs are associated with higher translation rates [67]. Therefore, it is conceivable that the translational preference for the shorter transcripts will be exacerbated as part of a detrimental cycle where more ribosomes and metabolic energy are produced. This would in turn lead to a bias against long transcripts with slower initiation scanning and translation rates. This hypothesis agrees with previous analyses that increase of translation-related genes may occur earlier during development which causes subsequent translational dysregulation of genes in ASD [58, 59].

Although exaggerated eIF4E function is related to ASD, the affected transcript targets of eIF4E that contribute to ASD pathology have not been fully understood. It is known that MNK1/2 kinase phosphorylates eIF4E at Ser209, promoting its binding to eIF4G for translation initiation [68]. By transiently overexpressing an active, phosphomimetic S209D mutant of eIF4E, we demonstrated that activation of eIF4E leads to increase of both cytoplasmic and mitochondrial translation. Particularly, translation of many ribosomal and mitochondrial transcripts was stimulated by eIF4E activation. This role of eIF4E was verified by the preferential reversal of ribosomal and mitochondrial mRNA translation when eIF4E S209-phosphorylation was inhibited by eFT508 in the VPA offspring. In line with our findings, eIF4G1 also promotes the synthesis of mitochondrial proteins involved in energy metabolism, thus playing a pivotal role in early neurodevelopment [69]. Additionally, MIA-induced offspring, which show upregulated eIF4E, also exhibit an increase of respiratory capacity and mitochondrial mass in the PFC neurons [25, 70]. Increased metabolic and mitochondrial gene expressions were also detected in TSC patient brain samples and derived-cells in which eIF4E S209-phosphorylation are elevated [26, 52]. Moreover, reanalysis of the transcriptome data of male *ADNP*^+/-^ mice, with higher eIF4E expression, reveals enhanced mitochondrial RNA metabolic process (Fig. S12), whereas female *ADNP*^+/-^ mice, without similar eIF4E change, exhibit reduced expression of the mitochondrial-encoded mt-Atp6 transcript [24, 71–73]. These results highlight the role of eIF4E in contributing to mitochondrial dysfunction in ASD. Increase mitochondrial respiration was also reported in neurons deficient with ASD risk genes *Taok2* (*Tao kinase 2*), *Syngap1*, or *Etfb* (*Electron-transfer-flavoprotein, beta polypeptide***)** [74]. It would be of interest to investigate whether eIF4E directly participates in or has crosstalks with molecular events mediated by these genes.

Various approaches attempting to reduce eIF4E activity have shown rescue effects of ASD-like features in *Fmr1* KO and *Nlgn3* KO mice. These include genetic reduction of MNK1/2 expression, pharmacological treatment of MNK1/2 inhibitors, knocking in a non-phosphorylatable form of eIF4E (S209A), and pharmacological treatment of an eIF4E-eIF4G interaction inhibitor [22, 27–30]. Moreover, MNK1/2 inhibitors also exhibit versatile potentials for treating cancer, pain, and depression [75]. In this study, we selected a brain available, highly selective MNK1/2 inhibitor eFT508 to investigate the effect of eIF4E inhibition in VPA-exposed offspring. We demonstrated that administration of eFT508 during juvenile, when eIF4E hyperphosphorylation first emerges, effectively corrected the social deficit and stereotyped behavior in VPA model mice. Moreover, this treatment regime showed long-lasting effect in reversal of core ASD-like behavioral deficits until adult, suggesting that the juvenile stage is a critical time window for targeted intervention against eIF4E overactivation. Nonetheless, we observed that eFT508 did not alleviate anxiety. We learned from the translatome result that eFT508 did not fully rescue the length-dependent alteration of the translatome in VPA offspring. Rather, it preferentially reversed the upregulated short metabolic transcripts in the VPA mice while had minimal effect to transcripts longer than 1 kb. Therefore, eFT508 may not exert direct effects on rescuing the synapse-related mRNA translation in the VPA offspring. Given the complexity of synaptic protein regulation, other mechanisms should be taken into consideration for the study of synapse gene translation in ASD.

In all, this study demonstrates a causal chain whereby elevation of eIF4E phosphorylation drives a length-dependent translatome imbalance that favors translation of ribosomal and mitochondrial mRNAs in the VPA-exposed offspring, leading to mitochondrial dysfunction and ASD-like behavioral deficits. Treatment of the MNK1/2 inhibitor eFT508 during juvenile exhibits a notable long-lasting effect on the reversal of translational alterations and core ASD behavioral deficits in VPA-exposed offspring. Overall, this study reveals a novel mechanism linking environmental and genetic factors of ASD and provides new evidence for research on the pathophysiology of protein synthesis perturbation in ASD.

## Materials and methods

### Mice

C57BL/6 mice, purchased from SPF (Beijing) Biotechnology Co., were used in all experiments. Mice were group-housed (four per cage) and given free access to water and food under controlled environmental conditions at 24 ± 2 °C with 50% relative humidity, and on a 12 h-light/dark cycle with light on from 08:00 to 20:00. All experimental procedures involving the use of animals were approved by the Ethics Committee on Animal Experiments at Jinan University. All animal experiments were performed according to the *Guide for the Care and Use of Laboratory Animals*. No formal randomization was used, and no animals were excluded. For drug treatment, mice were first tested for social interaction and weighed. Animals were then stratified based on sociability and body weight, and randomly assigned to drug or vehicle groups within each stratum.

VPA-exposed ASD mice model was established as follows: VPA (Sigma-Aldrich, P4543, St. Louis, MO, USA) was dissolved in 0.9% saline to a concentration of 50 mg/mL. A single dose of VPA (500 mg/kg, i.e., 10 µL/g body weight) was intraperitoneally (i.p.) injected into pregnant mice on gestational day 12.5. Equal volume of normal saline (10 µL/g body weight) was injected as the vehicle control. For behavioral tests and Western blotting on eIF4E phosphorylation, both male and female offspring from the same litters were tested. For other tests, only male mice were used.

### Antibodies

For Western blotting, the following antibodies were purchased from Cell Signaling Technology (Danvers, MA, USA): 4E-BP1 (9452S, 1:2000), p-Thr37/46-4EBP1 (p-4EBP1, 2855, 1:2000), 4E-BP2 (2845S, 1:1000), eIF4E (9742, 1:1000), p-Ser209-eIF4E (p-eIF4E, 9741S, 1:1000), ERK1/2 (4695S, 1:1000), p-Thr202/Tyr204-ERK (p-ERK, 4370, 1:1000), MNK1(2195S, 1:2000), p-Thr197/202-MNK (p-MNK1, 2111S, 1:2000), mTOR (2972S, 1:1000), p-Ser2448-mTOR (p-mTOR, 2971S, 1:2000), p70S6K (9209S, 1:1000), p-Thr389-p70S6K (p-70S6K, 9205S, 1:1000), RPS6 (2217S, 1:1000), p-Ser235/236-RPS6 (p-RPS6, 2211S, 1:1000), HSP90 (4877, 1:1000). Antibodies against α-tubulin (A01080, 1:10000) and β-actin (A01010, 1:10000) were from Abbkine Scientific (Atlanta, GA, USA), puromycin (MABE343, 1:2000) from Millipore Corporation (Billerica, MA, USA), PSD95(ab2723, 1:1000) from Abcam (Cambridge, UK), Synaptophysin (S5768, 1:1000) from Sigma-Aldrich, and GAPDH (20035, 1:10000) from ProMab Biotechnologies (Richmond, CA, USA).

The antibodies used for immunofluorescence were as follows: p-eIF4E (NBP3-21524, 1:150) from Novus Biologicals (Centennial, CO, USA), microtubule-associated protein 2 (MAP2; ab5392, 1:200) and PSD95 (ab2723, 1:200) from Abcam, and puromycin (MABE343, 1:200) from Millipore. The secondary antibodies Alexa Fluor 488 goat anti-mouse IgG antibody (A11029, 1:200) and Alexa Fluor 546 goat anti-Rabbit IgG antibody (A11035, 1:200) were from Invitrogen (Thermo Fisher Scientific Inc, Carlsbad, CA, USA).

### Behavioral tests

Social discrimination test was performed at juvenile stage (4–5 weeks old). Three-chamber test, self-grooming, and Open-field test were performed in male and female offspring at both 5 and 9 weeks old. Cognitive function tests and elevated Zero-maze test were performed only in male offspring in adulthood (9 weeks old). Mice were transferred to the behavior testing room to acclimate the environment 1 h before each experiment. In both the social discrimination test and Three-chamber test, all the unfamiliar mice were age- and sex-matched with the testing mice. The ambient illumination was 100–120 lux for the Open field test, and 40–60 lux for all other behavioral tests. All behavioral tests were recorded by an overhead video camera and analyzed by TopScan 3.0 (Clever Sys Inc, Washington, D.C., USA) [76, 77]. The experimenter was blinded to the groups of experiments.

#### Social discrimination test

Social discrimination test was conducted in a 40 cm (length) × 40 cm (width) × 40 cm (height) rectangular box. In the first phase, an empty wire cup was placed on one side of the arena, and the test mouse was allowed to freely explore the area for 10 min. In the second phase, a stranger mouse was placed in the cup, and the test mouse was allowed to explore freely again for 10 min. The duration of interaction with the empty cup or the stranger mouse were analyzed. The interaction index was calculated as the ratio of interaction time with the stranger mouse to interaction time with the empty cup.

#### Open field test and self-grooming analysis

The test mouse was put in the center of a 40 cm (length) × 40 cm (width) × 40 cm (height) arena and allowed to explore freely for 30 min [76, 77]. The travel distance and duration in the peripheral and center regions of the mice were analyzed. Additionally, the duration of grooming time of each mouse within the last 20 min of the 30-min test was analyzed. Grooming behaviors including washing face, licking paws, scratching or rubbing head and ears were counted.

#### Three-chamber test

Three-chamber test was carried out in a 60 cm (length) × 40 cm (width) × 20 cm (height) box with three chambers partitioned by two walls, each has a passage (5 × 5 cm) at the bottom [76, 77]. Following a 10-min habituation in the three empty chambers, the test mouse was subject to a 10-min social approach phase, in which it was allowed to explore an empty wire cup placed in one side chamber and a stranger mouse-containing wire cup in the other side chamber. The test was concluded with a 10-min social novelty phase, in which the test mouse was allowed to explore a familiar mouse-containing cup or an unfamiliar mouse-containing cup. Travel distance, the exploration time in different chambers, and sniffing time for the stranger mouse or empty cup were analyzed.

### Cell culture, plasmids construction, and transfection

The Neuro-2a and HEK293T cell lines, obtained from the American Type Culture Collection (ATCC), were maintained in Dulbecco’s Modified Eagle’s Medium (DMEM, Gibco, C11995500BT) supplemented with 10% Fetal Bovine Serum (Yeasen, 40130ES76) and 1% Penicillin-Streptomycin (Gibco, 15140122), at 37 °C in 5% CO_2_. The plasmids pCDH-3×FLAG-WT-eIF4E, pCDH-3×FLAG-eIF4E S209A (non-phosphorylatable), and pCDH-3×FLAG-eIF4E S209D (phosphomimetic) were constructed using standard cloning and mutagenesis method. Different plasmids were transfected in cells with 80% confluency using Lipofectamine LTX (Invitrogen, 15338100) following the manufacturer’s instruction.

### De novo protein synthesis measurements

For cortical protein synthesis measurement at E18.5, BONCAT was performed by i.p. injection of 50 μg/g AHA (Click Chemistry, 1066-100), a bioorthogonal analogue of methionine, to the pregnant mice at E17.5. After 16 h, embryos were harvested and the AHA-incorporated nascent proteins were coupled with disulfide biotin-DBCO (Click Chemistry Tools, A112) by copper free catalytic azide alkyne cycloaddition reaction. Streptavidin-Dynabeads (Thermo Fisher, 11206D) were used to pulldown the biotin-coupled nascent proteins, which was measured by Western blotting using HRP-conjugated streptavidin (Thermo Scientific, N100).

For measurement at postnatal stages, mice at P12 and P35 were sacrificed, and the brains were rapidly perfused to prepare slices as described previously [77]. The brain slices (300 μm) were placed in artificial cerebrospinal fluid (ACSF; 124 mM NaCl, 3 mM KCl, 1.25 mM NaH_2_PO_4_, 1 mM MgCl_2_, 2 mM CaCl_2_, 26 mM NaHCO_3_, and 10 mM glucose) and maintained at 32 °C for 40 min, bubbled with 95% O_2_ and 5% CO_2_. The slices were then transferred to bubbled ACSF containing 10 μg/ml puromycin (Sigma-Aldrich, P8833) for 25 min. Following the incubation period, the brain slices underwent two washes with ACSF, and the cortical and hippocampal regions were carefully separated for Western blotting using anti-puromycin antibody.

For puromycylation in cell cultures, Neuro-2a cells were transfected with plasmid for 24 h. Then puromycin (1 μM final concentration; Sigma-Aldrich, P8833) was added in the culture medium and incubated with the cells for 30 min. Cell lysates were then collected for Western blotting using anti-puromycin antibody. For in situ visualization of protein synthesis, AHA-based FUNCAT was used to visualize protein synthesis in the cytoplasm and mitochondria [69, 78]. Cells were incubated in a methionine-free medium, i.e., Dulbecco’s modified eagle medium without L-glutamine, L-methionine and L-cystine (Gibco, 21013024) supplemented with 200 μM L-cystine (MCE, HY-N0394) and 2 mM GlutaMAX (Gibco, 35050061), for 20 min to exhaust L-methionine taken up in the cells. Cells were then treated with 50 μM AHA (Invitrogen, C10102) for 30 min to label nascent proteins. To differentiate cytoplasmic and mitochondrial protein synthesis, 100 μg/mL of cycloheximide (Sigma, C7698) or both cycloheximide and 150 µg/mL chloramphenicol (MCE, HY-B0239) was pretreated the cells for 20 min before AHA was added. The cells were fixed, permeabilized, and AHA-labeled nascent proteins were covalently linked with Alexa Fluor 546 alkyne (Thermo Fisher, A10275) through a copper-catalyzed azide-alkyne cycloaddition (CuAAC) click reaction using the Click-iT Cell Reaction Buffer Kit (Invitrogen, C10269). FUNCAT signals were imaged using a Zeiss LSM 800 confocal microscope (Carl Zeiss AG).

### Polysome profiling

Polysome profiling of cortical tissues was performed through a continuous 20% to 50% sucrose density gradient via ultracentrifuge with SW-4Ti rotor (Beckman, Optima XPN-100), following the protocol published previously [79]. For Neuro-2a cells, samples were lysed immediately on ice in a buffer containing 15 mM Tris-HCl pH 7.5, 300 mM NaCl, 15 mM MgCl_2_, 1% Triton-X100 (v/v), 0.5% Na-deoxycholate (w/v), 100 U/mL RNase inhibitor (Thermo Fisher Scientific, Waltham, MA USA), 20 mM DL-Dithiothreitol (Aladdin Holdings Group Co., Ltd, Los Angeles, CA, USA), 0.1 mg/ml cycloheximide, 0.15 mg/mL chloramphenicol, and 1×protease inhibitor (TargetMol Chemicals Inc, Wellesley Hills, MA, USA). Lysates were separated through a continuous 10% to 50% sucrose density gradient via ultracentrifuge. 15 gradient fractions from light to heavy sedimentation were sequentially collected by a Piston Gradient Fractionator (BioComp Instruments, Nova Scotia, Canada) while RNA was detected at UV absorbance 260 nm. The peak areas of different ribosome components were quantitatively calculated by OriginPro 9.0 software (OriginLab Co., Northampton, US).

### RNA sequencing and translatome analysis

The samples obtained by polysome profiling were divided into non-poly fractions, containing non-translated or weakly translated mRNAs, and poly fractions, containing actively translated mRNAs. Total RNAs from non-poly and poly fractions, as well as from total homogenates, were extracted using TRIzol reagent (Invitrogen, 15596026). RNA-sequencing was performed on the DNBSEQ-T7 sequencing platform, yielding approximately 6 G of raw data per sample which were subjected to quality control using the FastQC package. The *NanoTrans* analysis pipeline [80] was utilized to align the data with the GRCm39 mouse genome reference (NCBI Annotation Release 109; https://www.ncbi.nlm.nih.gov/datasets/genome/GCF_000001635.27/). For the translatome analysis, DEGs were identified using Xtail R package [47] based on the values calculated from the read counts of polysome mRNA over non-poly mRNA fractions. For transcriptome analysis, DEGs were identified from total RNA-seq data using DESeq2 R package [81]. DEGs were defined by |Log_2_FC| > 0.5 and p-value < 0.05. Volcano plots of DEGs were generated using the ggplot2 R package, and enrichment analysis for all DEGs was performed using the ClusterProfiler R package, referencing the Gene Ontology (GO) database. GSEA analysis using the ClusterProfiler R package, referencing the MSigDB GSEA database. The R version 4.2.3 was used in this study.

### SFARI gene classification

Genes associated ASD were curated from the SFARI GENE database (https://gene.sfari.org/, updated on July 7, 2025) using the following criteria: *Genes with* ≥ *10 supporting reports* (*n* = 472) and *genes reported to be* syndromic (*n* = 464), filtered out if there is the term “syndromic” in the description of genetic-category. Gene lists of these two classifications are provided in Table S5 and S6.

### RNA extraction and quantitative real-time PCR (RT-qPCR)

RT-qPCR was conducted following the methods described previously [79]. cDNA synthesis from mRNAs in non-poly, poly, and total fractions was carried out using Moloney murine leukemia virus reverse transcriptase (MMLV-RT, Promega, M170A). qPCR amplification was performed using iQSYBR® Green Supermix (Bio-Rad, B21202) on a qPCR real-time system (Roche Light Cycler 480 Instrument). Primers for each gene were designed to amplify all possible spliced and edited variants, with the sequences listed as follows (from 5’ to 3’):

*mt-Nd1*-F: GGCCCATTCGCGTTATTCTT

*mt-Nd1*-R: AGCGTGGATAAGATGCTCGG

*mt-Nd5*-F: AACCAGCATTCCAGTCCTCAC

*mt-Nd5*-R: GTTGAGGTGGATTTTGGGATGG

*mt-Co1*-F: CTACCCACCTCTAGCCGGAA

*mt-Co1*-R: TGTGTTTAGGTTGCGGTCTGT

*mt-Co2*-F: GCTCTCCCCTCTCTACGCAT

*mt-Co2*-R: AGCAGTCGTAGTTCACCAGG

*mt-Cytb*-F: CCTTGACCCGATTCTTCGCT

*mt-Cytb*-R: GGGTGGGGTGTTTAGTGGAT

*mt-Atp6*-F: CGCCTAATCAACAACCGTCTC

*mt-Atp6*-R: CCAGCTCATAGTGGAATGGCT

*mt-Atp8*-F: GCCACAACTAGATACATCAACATGA

*mt-Atp8*-R: TGTTGGGGTAATGAATGAGGCA

*Fis1*-F: GAGGGCTGTTGCAGACTGAG

*Fis1*-R: CCAGTCCAATGAGTCCAGCC

*Timm13*-F: GGCTTCGGCTCGGATTTTG

*Timm13*-R: CACTTGTCCGTCATTCTCTGG

*Tomm6*-F: AGGTTCCAGACAACGTGGGA

*Tomm6*-R: AATGTCACTCAAGTTCCTGGC

*Ndufv3*-F: ATGTCTGTGAACGGAGCGG

*Ndufv3*-R: AGCACACTTTGTGTCTTGGGA

*Ndufaf8*-F: ATGTCTGTGAACGGAGCGC

*Ndufaf8*-R: GCGAACGCAGAGGTCCTTAC

*Rps12*-F: GCCAAAGCCTTAGACAAGCG

*Rps12*-R: GGCCTACCCATTCCCCTAGT

*Rps21*-F: GCCGAGGTTGATAGGACCAC

*Rps21*-R: ATCAGCCTTAGCCAATCGGAG

*Rps29*-F: GTCTGAAGGCAAGATGGGTCA

*Rps29*-R: TGGTAGTAGTCAGTCGAATCCA

*Rpl3l*-F: GAAGGGCCGGGGTGTTAAAG

*Rpl3l*-R: AGCTCTGTACGGTGGTGGTAA

*Rpl8*-F: AAGGCGCGGGTTCTGTTTT

*Rpl8*-R: GCTCTGTCCGCTTCTTGAATC

*Rplp1*-F: CAAGGCTCTGGCCAATGTCA

*Rplp1*-R: AGTCAAAAAGACCGAAGCCCA

*Mrps26*-F: CATGACCCGCCTGCCAAAT

*Mrps26*-R: TCTCCCGGTACTGTCGGTAG

*Mrpl28*-F: CCTCACGGGACCAAGTTCAA

*Mrpl28*-R: CCCTCTTGGAGAGCTTGTCG

### LC-MS/MS and data analysis

Cerebral cortices were homogenized in ice-cold lysis buffer containing 320 mM sucrose, 5 mM Tris-HCl (pH 7.4), 1 mM MgCl_2_, 1 mM DTT, and protease and phosphatase inhibitor cocktail (TargeMol, C0004). The homogenate was centrifuged at 1000×*g* for 10 min at 4 °C to remove debris and nuclei. The supernatant was then centrifuged at 13000×*g* for 15 min at 4 °C, and the pellet (crude synaptosome fraction) was suspended in a solution containing 320 mM sucrose, 5 mM Tris-HCl (pH 7.4), and protease and phosphatase inhibitors. Proteins were quantified using BCA assay kit (Thermo Fisher, 23252). 80 μg of protein per sample was processed on the PreOn platform using the iST Protein Preparation Kit (PreOmics, P.O.00027, Germany), with trypsin digestion for 3 h. Digested peptides were desalted, vacuum-dried, and reconstituted in 0.1% formic acid for quantification. A total of 300 ng of peptides was loaded onto a nanoElute2 system coupled to a timsTOF HT (Bruker Corporation, USA) via CaptiveSpray ionization. Peptides were separated using a 55-min linear gradient from 2% to 37% acetonitrile (in 0.1% formic acid). Data were acquired in diaPASEF mode (m/z 300–1,500). Database searches were performed in PEAKS Studio 12 against the SwissProt *Mus musculus* database (17,214 entries). Parameters included trypsin specificity, up to 2 missed cleavages, peptide length 7–30 residues, variable modifications (methionine oxidation, N-terminal acetylation), and fixed carbamidomethylation. Match-between-runs, label-free quantification, and total ion current normalization were applied. Proteins with at least one unique peptide detected were identified and quantified. Differentially expressed proteins were defined as up-regulated (fold change > 1.1, *p* < 0.05) or down-regulated (fold change < 0.9, *p* < 0.05).

### ATP assay

ATP levels were measured using an ATP Assay Kit (Beyotime, S0026). Cortical tissues were immediately homogenized in the ATP detection lysis buffer, centrifuged at 4 °C at 12000×*g* for 5 min, and the supernatant was collected for the measurement of ATP content using Infinite F500 (TECAN, Switzerland).

### Transmission electron microscopy (TEM) and image processing

Mice were euthanized and the cerebral cortices were rapidly dissected on ice. The mPFC was micro-dissected out and immediately fixed in the TEM fixative solution for 2 h at room temperature before placed at 4 °C. Subsequent steps for routine TEM, including dehydration, sectioning, and imaging, were carried out. Sections were imaged using a Hitachi TEM system at 80.0 kV. Analyses of the number and morphology of mitochondria within the synaptic areas, the number of presynaptic vesicles, and the morphology of synapses were performed using ImageJ software (National Institutes of Health, Bethesda, MD, United States).

### Mitochondrial complex activity assays

Different mitochondrial complex activity levels were measured using the following kits correspondingly: Mitochondrial Complex I Activity Assay Kit (Elabscience, E-BCK149-M), Mitochondrial Complex II Activity Assay Kit (Elabscience, E-BCK150-M), Mitochondrial Complex III Activity Assay Kit (Elabscience, E-BCK151-M), Mitochondrial Complex IV Activity Assay Kit (Elabscience, E-BCK152-M), and Mitochondrial Complex V Activity Assay Kit (Elabscience, E-BCK153-M). Briefly, cortical tissue was homogenized and centrifuged at 600×*g* for 5 min at 4 °C, and the supernatant was then centrifuged at 11000×*g* for 10 min at 4 °C. The pellet was resuspended and sonicated for 1 min at 4 °C, and centrifuged at 11000×*g* for 10 min at 4 °C. The supernatant was collected for the measurement of different mitochondrial complex activities.

### Primary cortical neuron culture from VPA-exposed embryos

A single dose of VPA (500 mg/kg body weight) was i.p. injected in pregnant C57BL/6 mice on gestational day 12.5, and normal saline was injected as the vehicle control. Primary cortical neurons were cultured from VPA- or saline-exposed embryos at E17.5 following a protocol published previously [82, 83]. Briefly, the cortical tissues were trypsinized to obtain a single-cell suspension, and then seeded on 18-mm diameter coverslips coated with 1 mg/mL poly-D-lysine (Millipore, A-003-E) at a density of 2.5×10^4^ cells per coverslip, and cultured in Neurobasal Medium (Gibco, 21103049) supplemented with 2% B27 (Gibco, 17504044), 1 mM L-Glutamine (Gibco, 25030081), and 0.18% D-Glucose (Gibco, A2494001). Half of the medium was changed every three to four days.

### Mitochondrial membrane potential assay

Mitochondrial membrane potential in primary cortical neurons was measured using the Mitochondrial Membrane Potential Assay Kit with TMRM (Beyotime, C2001S). Briefly, TMRM was diluted to 1×solution and added to primary cortical neurons at 15 days in vitro (DIV) for 30 min at 37 °C. Carbonyl cyanide m-chlorophenyl hydrazone (CCCP), a mitochondrial membrane potential depolarization agent, was added to cells at a concentration of 10 μM for 20 min pre-incubation, acting as a negative control. After incubation, the cells were washed twice and TMRM fluorescence was photographed in live cells using Zeiss LSM 800 confocal microscope.

### Immunofluorescence

To analyze dendritic PSD95 puncta, primary cortical neurons at 14 DIV were fixed and immunostained with mouse anti-PSD95 and rabbit anti-MAP2 antibodies as previously described [84]. Images were acquired using Zeiss LSM 800 confocal microscope with a 63× objective (Carl Zeiss AG). PSD95 puncta density was quantified in MAP2-positive dendrites using ImageJ software.

To analyze the levels of p-eIF4E and puromycin in the cortex, cryosections (20 μm) were made using a frozen microtome (Leica, CM1950). The sections were permeabilized with 0.3% Triton X-100 in PBS for 30 min, then blocked with 3% bovine serum albumin (Solarbio, A8020) and 10% goat serum (GENOM, GNMGS100) at 37 °C for 1 h. The sections were then incubated with rabbit anti-p-eIF4E and mouse anti-puromycin antibodies at 4 °C overnight, followed by 1 h incubation at room temperature. Secondary antibodies Alexa Fluor 488 goat anti-mouse IgG and Alexa Fluor 546 goat anti-rabbit IgG were applied at 37 °C for 1 h. Nuclei were counterstained with 4’,6-diamidino-2-phenylindole (DAPI; Sigma-Aldrich, D9542, 1:1000) for 10 min. Sections were mounted with Antifade Mounting Medium (Beyotime, P0128S) and imaged on Zeiss LSM 800 confocal microscope.

### Mitochondrial respiratory chain enzyme analysis

The Oroboros O2k experiment was conducted following an established protocol with the Oroboros O2k platform (Oroboros Instruments, Austria) [85]. HEK293T cells were plated in 6-well plates and detached by treating with a 0.05% trypsin-EDTA solution (Gibco, 15400054) for 30 s. Afterward, the cells were counted and adjusted to a density of 1×10^6^ cells per 100 µL. Only two samples were tested simultaneously. Basal respiration was recorded after 10-min equilibration at 37 °C. Oligomycin (5 nM final concentration; Sigma-Aldrich, O4876) was then added to inhibit ATP synthase (Complex V). Maximal respiration was induced by titrating carbonyl cyanide 4-(trifluoromethoxy)phenylhydrazone (FCCP; 1 µM final concentration; Sigma-Aldrich, C2920). Rotenone (0.5 µM final concentration; Sigma-Aldrich, R8875) was injected to inhibit Complex I. Antimycin A (2.5 µM final concentration; Sigma-Aldrich, A8674) was added to inhibit Complex III. Oxygen consumption rates were normalized to cell count and expressed as pmol O₂/s/10⁶ cells.

### Drug preparation and treatment

eFT508 (Tomivosertib; MedChemExpress LLC, Monmouth Junction, NJ, USA) was dissolved in DMSO at a concentration of 5 mg/mL as a stock solution. The working drug solution was made by freshly diluted the stock solution in normal saline with PEG300 (40% v/v) and Tween-80 (5% v/v) to a concentration of 1 mg/mL. The prenatally VPA-exposed male mice were randomly allocated to vehicle group (VPA-DMSO) and drug-treated group (VPA-eFT508), and mouse in each group received 4 times daily i.p injections of vehicle or eFT508 (1 mg/mL) correspondingly at postnatal week 3. Behavior tests including Open field test, self-grooming analysis, Three-chamber test, and elevated Zero-maze test were performed at both postnatal Weeks 4–6 and Week 9. A single drug dose was administered 1 h prior to each behavioral experiment and tissue collection.

### Statistical analysis

No a priori power analysis was conducted because the sample sizes for each experimental group were based on pilot data and published studies [76, 77, 79]. All experiments were conducted in a blinded manner. Statistical analyses were carried out using GraphPad Prism 9.2.0. The results were presented as mean ± SEM. Distribution normality (Shapiro–Wilk test) and homogeneity of variance of the data were checked before parametric analyses. No exclusion criteria were pre-determined in our tests. Differences between the two groups were assessed using two-tailed *t*-test, and multiple-group comparisons were analyzed using one-way analysis of variance (ANOVA) followed by post hoc Tukey’s multiple comparisons. Differences between distributions were compared using KS test or two-sample z test. *P*-value less than 0.05 was considered statistically significant for all statistical analyses. Detailed statistical information of each experiment was provided in Table S10.

## Supplementary information

Supplementary Information

Supplementary Table S1. Translational DEGs list (VPA vs. CTL)

Supplementary Table S2. Expressions of core regulators of mRNA Poly(A) tail modification (VPA vs. CTL)

Supplementary Table S3. GO analysis of translational DEGs (VPA vs. CTL)

Supplementary Table S4. GSEA analysis of the translatome data (VPA vs. CTL)

Supplementary Table S5. Syndromic gene list (n = 464) from SFARI database

Supplementary Table S6. List of Genes with XXX 10 supporting reports (n = 472) from SFARI database

Supplementary Table S7. Translational DEGs list (VPA-eFT508 vs. VPA-DMSO)

Supplementary Table S8. eFT508 reversed translational DEGs list (changed in VPA vs. CTL but reversed in VPA-eFT508 vs. VPA-DMSO)

Supplementary Table S9. GO analysis of eFT508 reversed translational DEGs (changed in VPA vs. CTL but reversed in VPA-eFT508 vs. VPA-DMSO)

Supplementary Table S10. Statistical Details

## Data Availability

The raw sequencing data generated in this study have been deposited in the Genome Sequence Archive (GSA) at the National Genomics Data Center (NGDC), China National Center for Bioinformation / Beijing Institute of Genomics, Chinese Academy of Sciences, under accession numbers CRA037712 and CRA037713. The data are associated with BioProject PRJCA056529 and are publicly accessible at https://ngdc.cncb.ac.cn/gsa/. The analysis codes of this study are available from the corresponding author upon reasonable request.
