# Supplementary material for: Correction of eIF4E overactivation rescues translatome imbalance and core ASD-like behaviors in valproic acid-induced offspring mice
Source: Mol Psychiatry. 2026 Mar 7;31(7):3987–4005. doi: 10.1038/s41380-026-03517-3 (PMC13269136; doi:10.1038/s41380-026-03517-3)
Supplement: Supplementary file 1 — Supplementary Information [file 41380_2026_3517_MOESM1_ESM.pdf]

Correction of eIF4E overactivation rescues translatome imbalance and core ASD- like behaviors in valproic acid-induced offspring mice

Miaoqi Huang, Han Ye, Yong Xu, Jiaoyan Xie, Xinyu Wang, Yan Luo, Peng Liu, Xuanyue Ma, Shiqing Zhang, Bin Jiang, Wen-Cai Ye*, Yinghui Peng*, Lei Shi*

Supplementary Information

Supplementary Figures

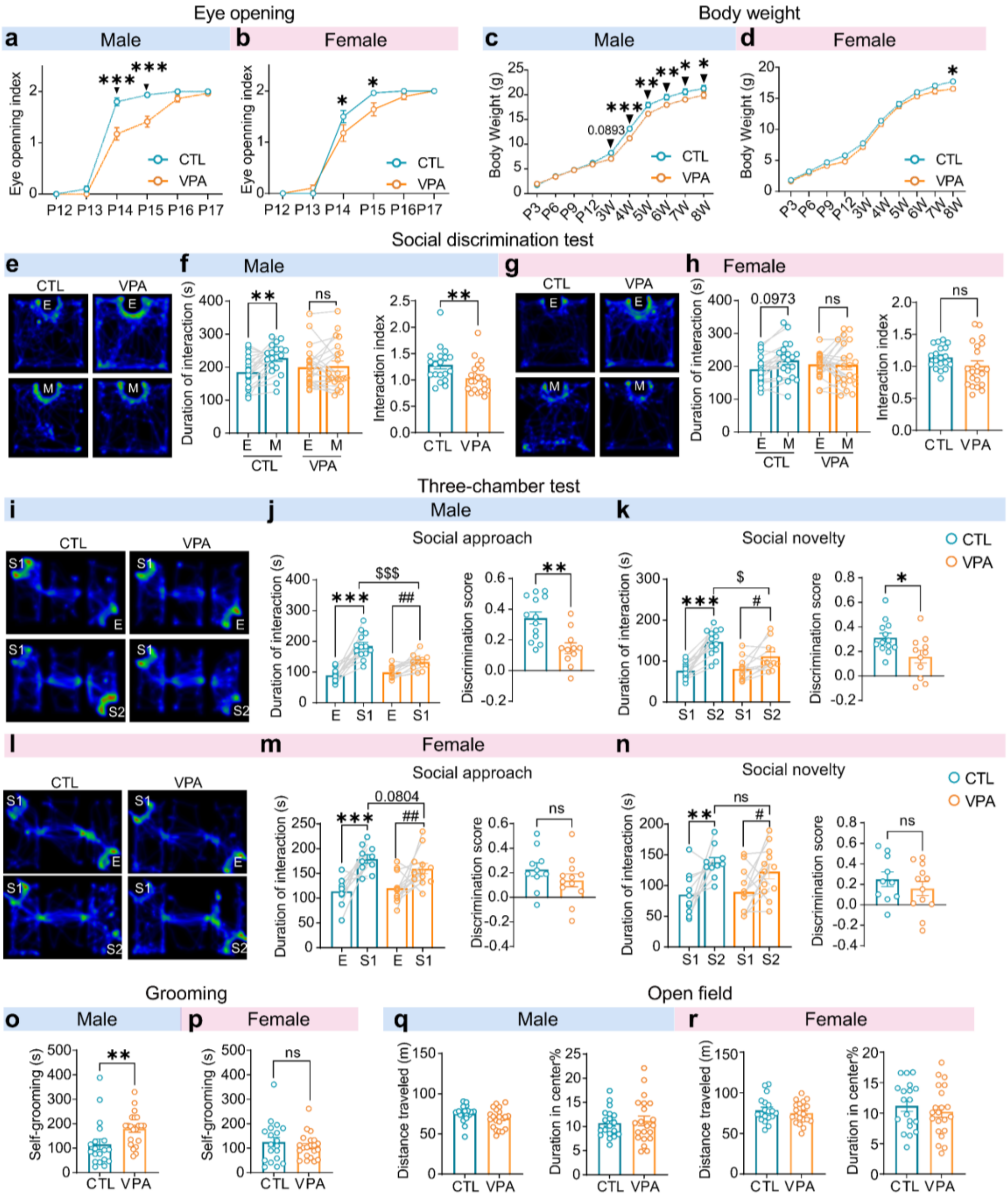

Figure S1. Comparisons of developmental delay and core ASD-like behavioral deficits in VPA-exposed male and female offspring.

(**a**) Eye-opening index of male control (CTL) and VPA-exposed (VPA) mice. CTL, $n = 30$ mice; VPA, $n = 29$ mice. Two-way ANOVA, ***$p < 0.001$.

(**b**) Eye-opening index of female CTL and VPA mice. CTL, $n = 28$ mice; VPA, $n = 28$ mice. Two-way ANOVA, *$p < 0.05$.

(**c**) Body weight trajectories of male CTL and VPA mice. CTL, $n = 24$ mice; VPA, $n = 22$ mice. Two-way ANOVA, *$p < 0.05$, **$p < 0.01$, ***$p < 0.001$.

(**d**) Body weight trajectories of female CTL and VPA mice. CTL, $n = 17$ mice; VPA, $n = 12$ mice. Two-way ANOVA, *$p < 0.05$, **$p < 0.01$, ***$p < 0.001$.

(**e**) Representative activity heatmaps of male CTL and VPA mice during Phase 1 (interacting with the empty cup; E) and Phase 2 (interacting with the stranger mouse; M) of the social discrimination test.

(**f**) Time spent interacting with E and M (left panel), and interaction index of M over E (right panel) of the male mice. CTL, $n = 19$ mice; VPA, $n = 21$ mice. Unpaired *t* test, CTL-M vs. E, **$p < 0.01$; VPA-M vs. E, not significant (ns); VPA vs. CTL, **$p < 0.01$.

(**g**) Representative activity heatmaps of female CTL and VPA mice during Phase 1 and Phase 2 of the social discrimination test.

(**h**) Time spent interacting with E and M (left panel), and interaction index of M over E (right panel) of the female mice. CTL, $n = 19$ mice; VPA, $n = 21$ mice. Unpaired *t* test, CTL-M vs. E, p =0.0973; VPA-M vs. E, ns; VPA vs. CTL, ns.

(**i**) Representative activity heatmaps of male CTL and VPA mice during social approach (upper panels) and social novelty (lower panels) stages of the Three-chamber test. E: empty cup; S1: stranger mouse #1; S2: stranger mouse #2.

(**j**) Time spent interacting with S1 and E (left panel), and discrimination score during social approach (right panel) of the male mice. CTL, $n = 13$ mice; VPA, $n = 11$ mice. Unpaired *t* test, CTL-S1 vs. E, ***$p < 0.001$; VPA-S1 vs. E, ##$p < 0.01$; VPA-S1 vs. CTL-S1, $$$$p < 0.001$; VPA vs. CTL, **$p < 0.01$.

(**k**) Time spent interacting with S2 and S1 (left panel), and discrimination score during social novelty (right panel) of the male mice. CTL, $n = 13$ mice; VPA, $n = 11$ mice. Unpaired *t* test, CTL-S2 vs. S1, ***$p < 0.001$; VPA-S2 vs. S1, #$p < 0.05$; VPA-S2 vs. CTL-S2, $$p < 0.05$; VPA vs. CTL, *$p < 0.05$.

(**l**) Representative activity heatmaps of female CTL and VPA mice during social approach (upper panels) and social novelty (lower panels) stages of the Three-chamber test.

(**m**) Time spent interacting with S1 and E (left panel), and discrimination score during social approach (right panel) of the female mice. CTL, $n = 10$ mice; VPA, $n = 12$ mice. Unpaired *t* test, CTL-S1 vs. E, ***$p < 0.001$; VPA-S1 vs. E, ##$p < 0.01$; VPA-S1 vs. CTL-S1, p =0.0804; VPA vs. CTL, ns.

(**n**) Time spent interacting with S2 and S1 (left panel), and discrimination score during social novelty (right panel) of the female mice. CTL, $n = 10$ mice; VPA, $n = 12$ mice. Unpaired *t* test,

CTL-S2 vs. S1, ***$p < 0.01$; VPA-S2 vs. S1; #$p < 0.05$; VPA-S2 vs. CTL-S2, ns; VPA vs. CTL, ns.

(**o**) Self-grooming duration of male CTL and VPA mice. CTL, $n = 19$ mice; VPA, $n = 21$ mice. Mann-Whitney test, *$p < 0.05$.

(**p**) Self-grooming duration of female CTL and VPA mice. CTL, $n = 19$ mice; VPA, $n = 21$ mice. Mann-Whitney test, ns.

(**q**) Travel distance and percentage of time spent in the center zone by male CTL and VPA mice in the Open-field test. CTL, $n = 19$ mice; VPA, $n = 21$ mice.

(**r**) Travel distance and percentage of time spent in the center zone by female CTL and VPA mice in the Open-field test. CTL, $n = 19$ mice; VPA, $n = 21$ mice.

Data are expressed as mean ± SEM.

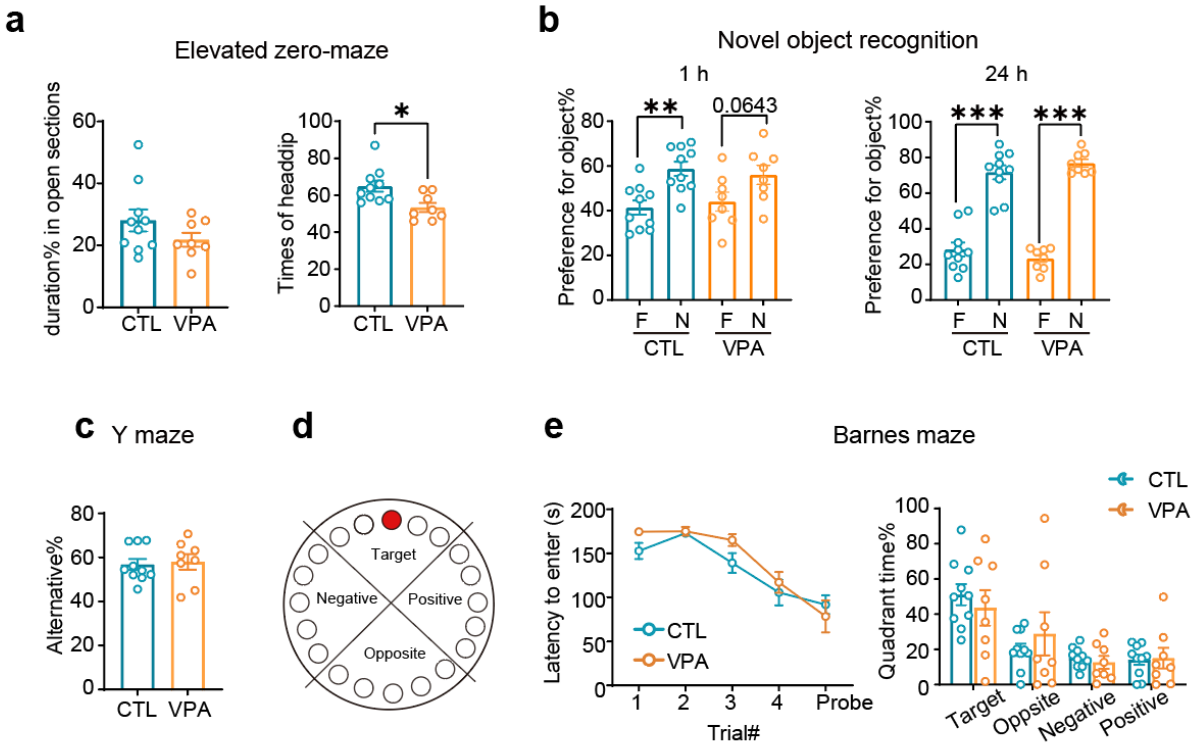

Figure S2. Mice with prenatal exposure of VPA display anxiety-like behavior but normal cognitive functions.

(**a**) Percentage of time spent in the open sections and number of head-dips by male CTL and VPA mice in elevated Zero-maze test. CTL, n = 10 mice; VPA, n = 8 mice. Mann-Whitney test, *p < 0.05.

(**b**) Percentage of exploration time for the novel (N) and familiar (F) object at 1 hour and 24 hours after training in the novel object recognition test. CTL, n = 10 mice; VPA, n = 8 mice. One-way ANOVA, **p < 0.01, ***p < 0.001.

(**c**) Spontaneous alternation rate of male CTL and VPA mice in the Y maze. CTL, n = 10 mice; VPA, n = 8 mice.

(**d**,**e**) Latency to enter and percentage of each quadrant time of male CTL and VPA mice in Barnes maze test. CTL, n = 10 mice; VPA, n = 8 mice.

Data are expressed as mean ± SEM.

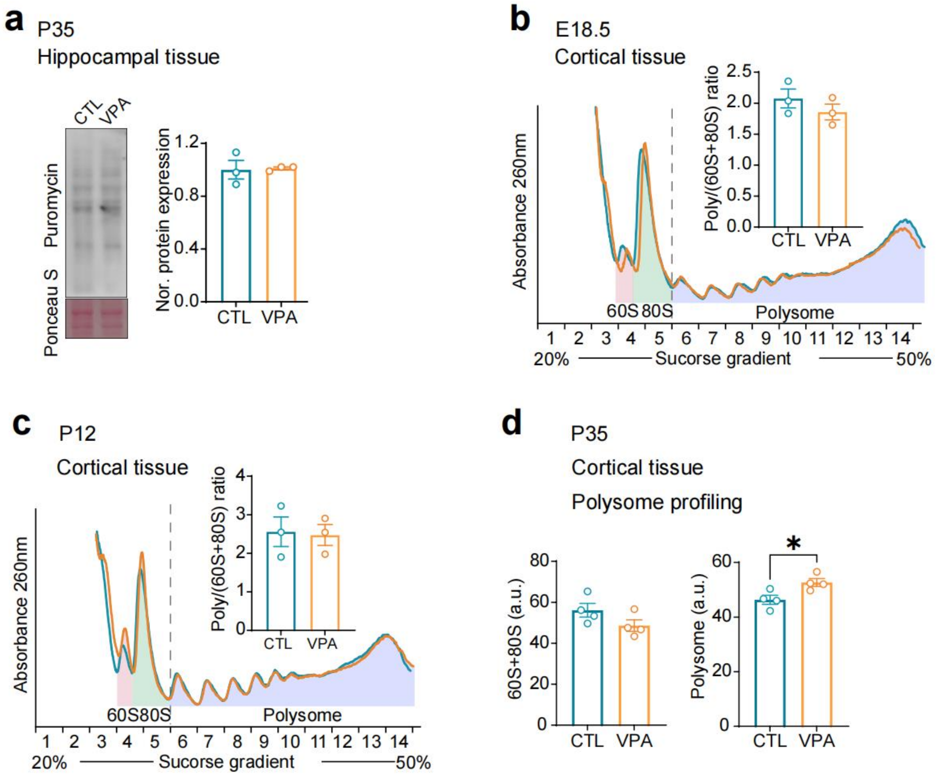

Figure S3. No change of hippocampal protein synthesis, but elevation of cortical protein synthesis was observed in the VPA-exposed offspring.

(**a**) Representative immunoblots (left panel) and quantification analysis (right panel) of puromycin-labeled de novo global protein synthesis in hippocampal tissues of CTL and VPA mice at P35. Ponceau S was used as a loading control. $n = 3$ mice for each group.

(**b,c**) Representative polysome profiling from the cerebral cortex of CTL and VPA mice at E18.5 (**b**) and P12 (**c**). The polysome/(60S+80S) ratio (embedded panels) was calculated as the ratio of polysome peak area over the sum of 60S and 80S peak areas from each profiling, correspondingly. $n = 3$ mice in each group.

(**d**) The quantification of the areas of 60S+80S and polysome peaks from polysome profiling in cortical tissues at P35. $n = 4$ mice for each group. Unpaired t test, $*p < 0.05$.

Data are expressed as mean ± SEM.

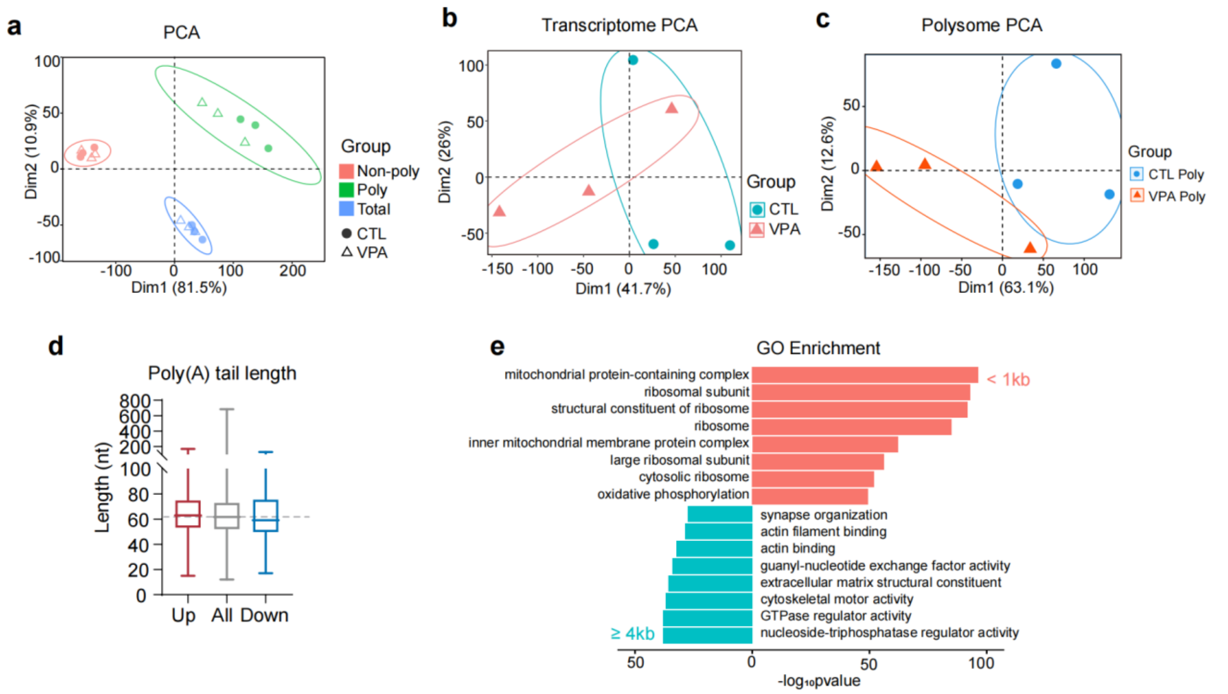

Figure S4. Integrated analysis reveals differential gene expression profiles in cerebral cortex of VPA mice.

(**a–c**) Principal Component Analysis (PCA) plot of CTL and VPA datasets in non-poly, poly and total groups (**a**), transcriptome (**b**), and polysome fractions (**c**).

(**d**) Box-whisker plot showing the poly(A) tail length of translational upregulated and downregulated DEGs, in comparison with those of all genes in the translatome. The dotted line represents the median value of all genes.

(**e**) Bubble plots showing GO enrichment result of short CDS transcripts (< 1 kp) and long CDS transcripts (⩾ 4 kp).

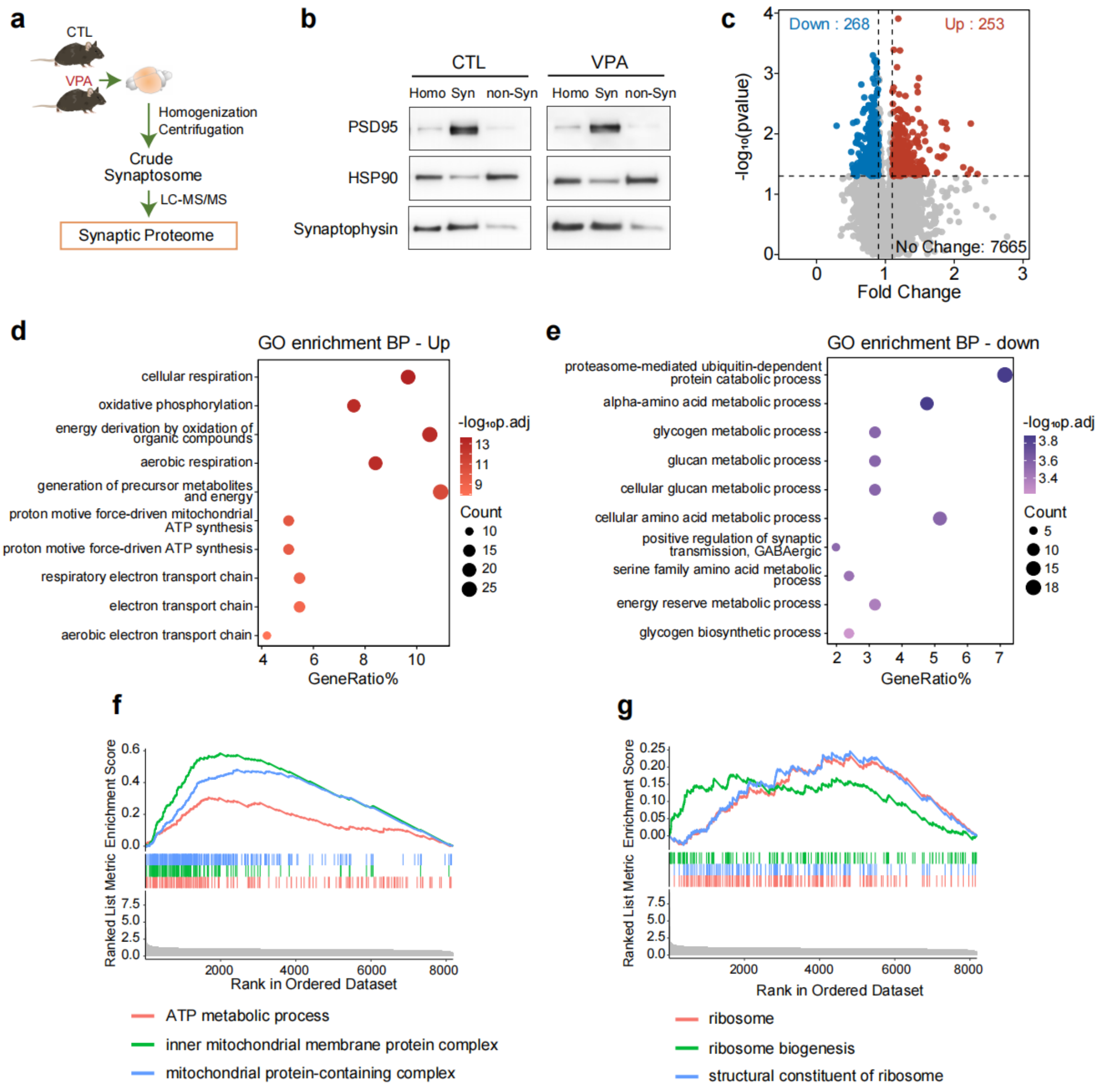

Figure S5. Synaptic proteome analysis in the cerebral cortex of VPA mice.

(**a**) Experimental schematic of synaptic proteomics.

(**b**) Representative immunoblots of total brain homogenate (Homo), crude synaptosome (Syn), and non-synaptosome (non-Syn) fractions.

(**c**) Volcano plot showing differentially expressed proteins in the cerebral cortex of VPA vs. CTL mice, with up-regulated proteins highlighted in red and down-regulated in blue. Up-regulated proteins were defined by fold change > 1.1 and p-value < 0.05, down-regulated proteins were defined by fold change < 0.9 and p-value < 0.05.

(**d**,**e**) Bubble plots showing biological processes (BPs) enriched in the GO analysis of upregulated and downregulated proteins.

(**f**) GSEA result showing upregulation of mitochondria-related gene sets (GO terms) including ATP metabolic process (NES = 3.143, FDR = 8.34E-08), inner mitochondrial membrane protein complex (NES = 5.496, FDR = 3.31E-09), and mitochondrial protein-containing complex (NES = 5.528, FDR = 3.31E-09). NES, normalized enrichment score.

(**g**) GSEA result showing upregulation of ribosome-related gene sets (GO terms) including ribosome (NES = 2.516, FDR = 0.0001), ribosome biogenesis (NES = 1.816, FDR = 0.0368), structural constituent of ribosome (NES = 2.397, FDR = 0.0005).

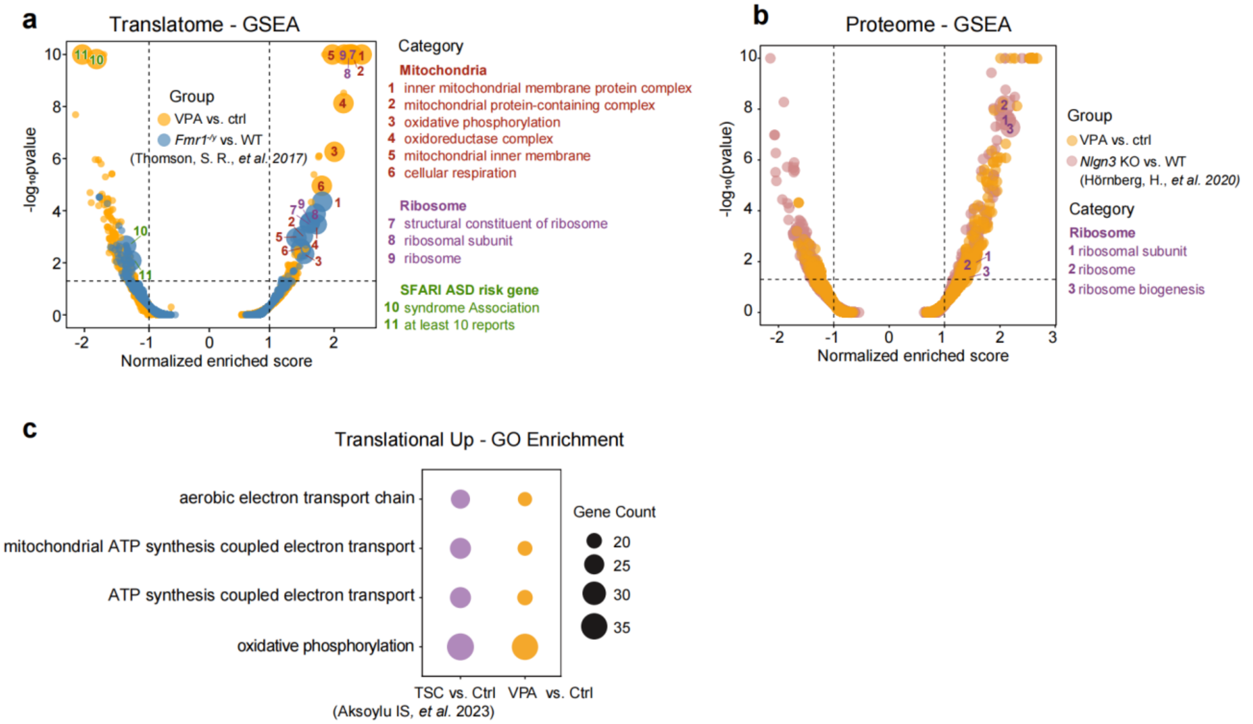

Figure S6. Common translation upregulation of ribosomal and/or mitochondrial genes in VPA-exposed mice and several genetic models of ASD.

(**a**) Volcano plot showing GSEA of translatome datasets of VPA mice in the current study and *Fmr1*$^{-/y}$ mice from Thomson, S. R., *et al* (50). A cutoff value with $p < 0.05$ and $|NES| \geq 1$ was used.

(**b**) Volcano plot of GSEA analysis of proteome datasets from VPA mice in the current study and *Nlgn3* KO mice from Hörnberg, H., *et al* (28). A cutoff value with $p < 0.05$ and $|NES| \geq 1$ was used.

(**c**) Bubble plot of GO analysis of upregulated gene in the translatome datasets from VPA mice in the current study and Tuberous sclerosis complex (TSC) patients from Aksoylu IS, *et al* (52). A cutoff value with adjust $p < 0.05$.

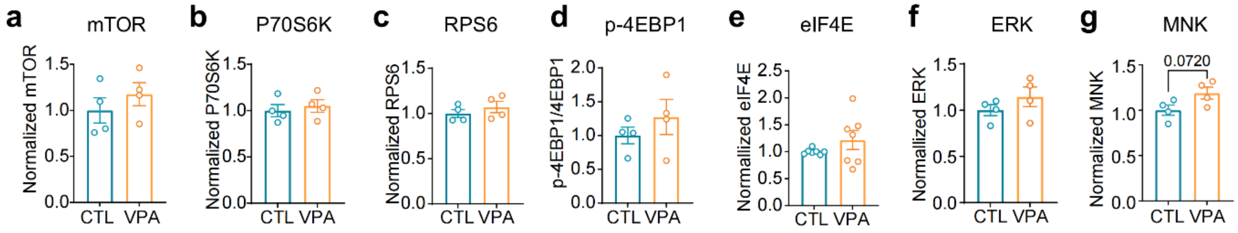

Figure S7. Effects on mTOR-p70S6K-RPS6 and MNK-eIF4E pathways in the cortex by prenatal VPA exposure.

(**a**–**g**) Quantification of protein expressions of different molecules within MNK-eIF4E and mTOR-p70S6K-RPS6 pathways in the cortex of VPA and control (CTL) cortex at P35. Unpaired *t* test, $p = 0.0720$. Data are expressed as mean ± SEM.

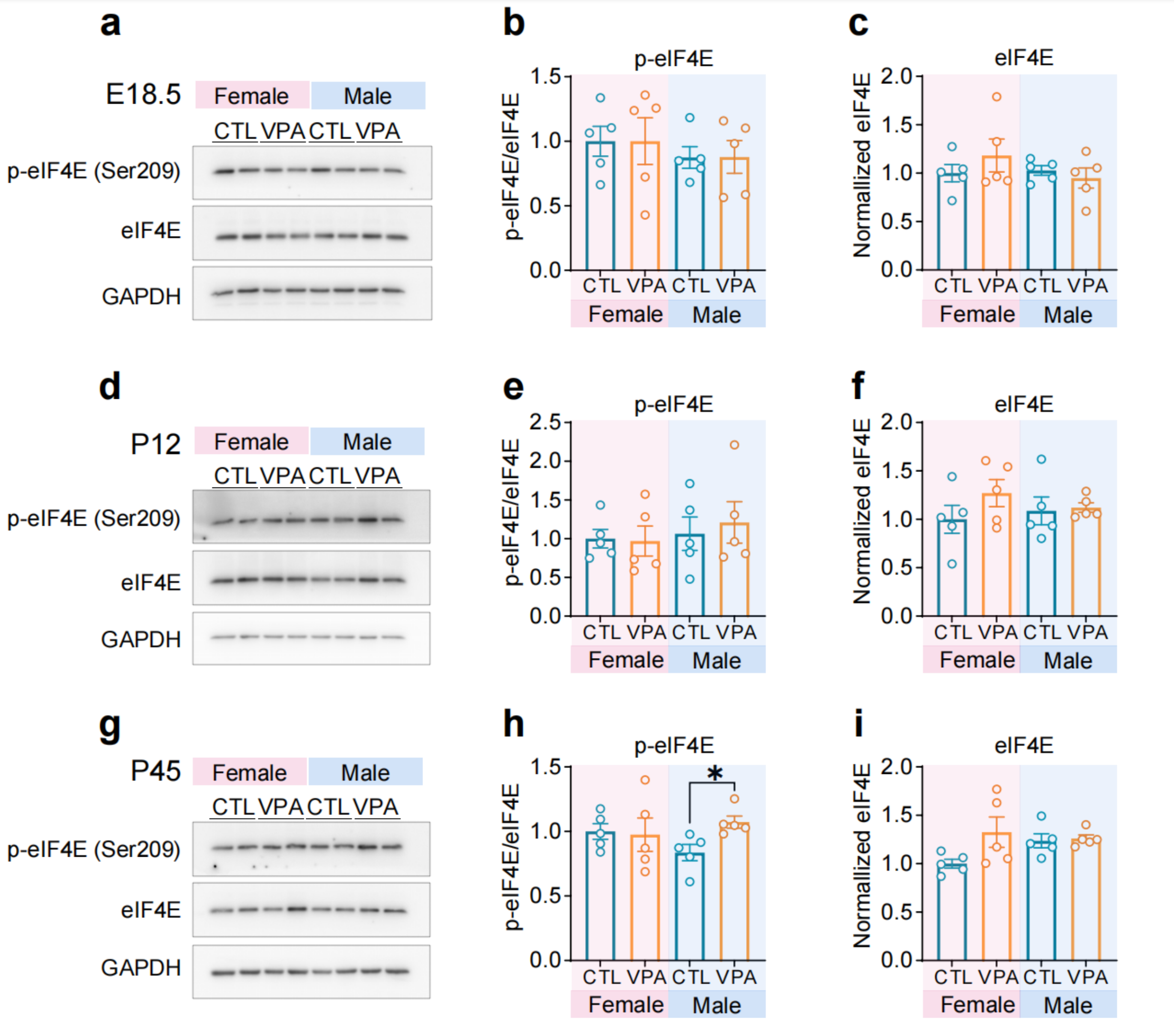

Figure S8. p-eIF4E (Ser209) levels in cortical tissues of VPA-exposed male and female offspring at different developmental stages.

(**a**–**i**) Representative immunoblots and quantification of p-eIF4E and total eIF4E in male and female offspring at E18.5, P12 and P45. $n = 5$ per group per sex. Unpaired t test, $*p < 0.05$. Data are expressed as mean ± SEM.

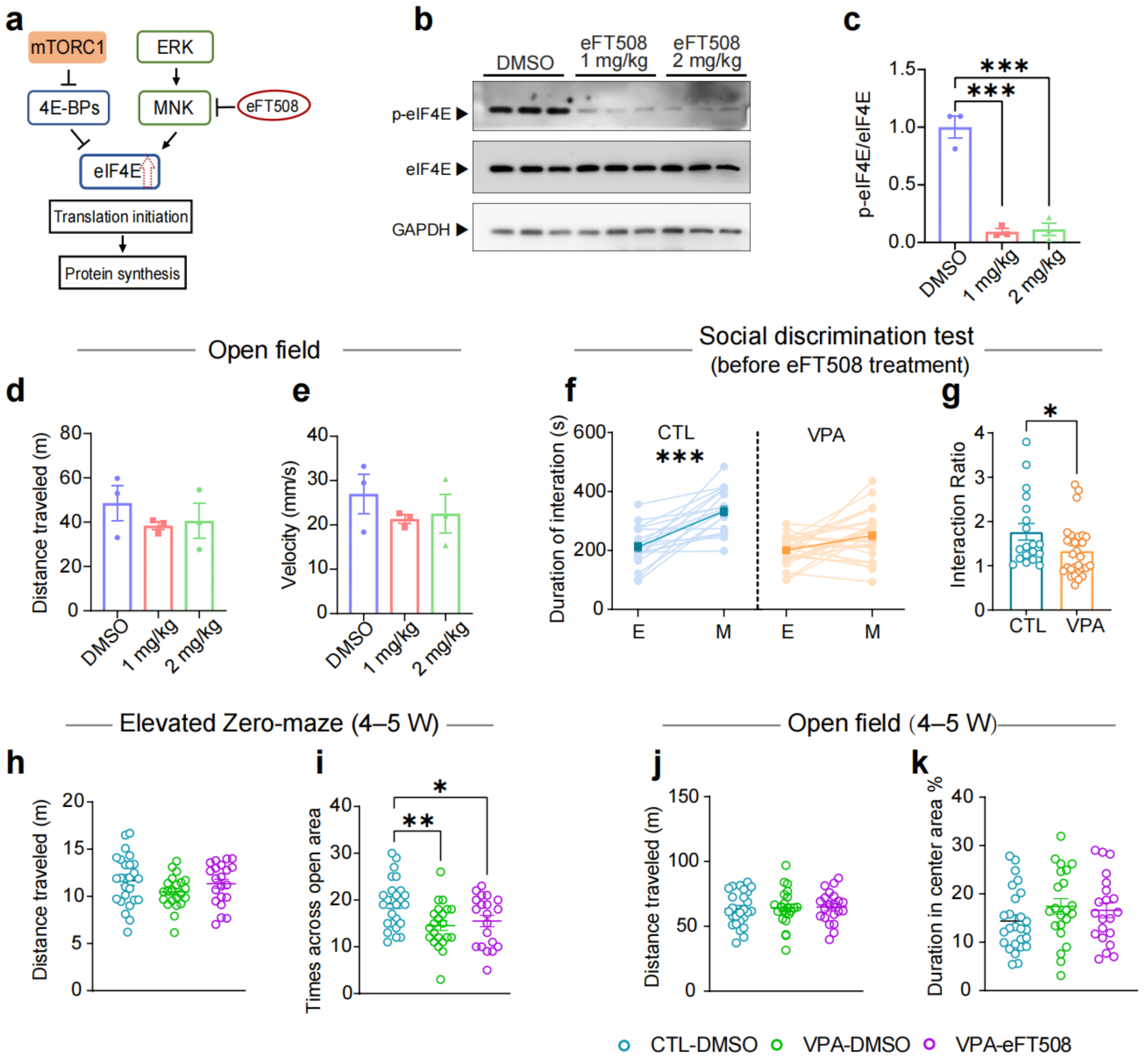

Figure S9. eFT508 inhibits eIF4E phosphorylation and exhibits no effects on mice performance in the Open-field and elevated Zero-maze test.

(**a**) Schematic illustration of the major signaling pathways regulating eIF4E activity and the acting site of eFT508.

(**b**,**c**) Representative immunoblots (**b**) and qualification (**c**) of p-eIF4E (Ser209) levels in the cerebral cortex of mice at P25 after eFT508 i.p. treatment for 1 h. GAPDH was used as a loading control. n = 3 mice for each group, One-way ANOVA, ***$p < 0.001$, 1 mg/kg or 2 mg/kg vs. DMSO (vehicle).

(**d**,**e**) Travel distance and velocity of mice in the Open-field test after eFT508 i.p. treatment for 1 h.

(**f**,**g**) Social discrimination performance of VPA mice before eFT508 treatment at P21. Interaction ratio is calculated by the ratio of time spent in sniffing stranger mouse (M) and empty cup (E). CTL, n = 19 mice; VPA, n = 27 mice. Unpaired t test, *$p < 0.05$, ***$p < 0.001$.

(**h,i**) Total distance traveled (**h**) and times across open sections (**i**) in the elevated Zero-maze test by CTL-DMSO, VPA-DMSO, and VPA-eFT508 mice at 4–5 weeks old. CTL-DMSO, n = 26 mice; VPA-DMSO, n = 22 mice; VPA-eFT508, n = 21 mice. One-way ANOVA, $*p < 0.05$, $**p < 0.01$, VPA-DMSO or VPA-eFT508 vs. CTL-DMSO.
(**j,k**) Total distance traveled (**j**) and percentage of duration in center area (**k**) by CTL-DMSO, VPA-DMSO, and VPA-eFT508 mice in the Open-field test at 4–5 weeks old. CTL-DMSO, n = 26 mice, VPA-DMSO, n = 22 mice, VPA-eFT508, n = 21 mice.

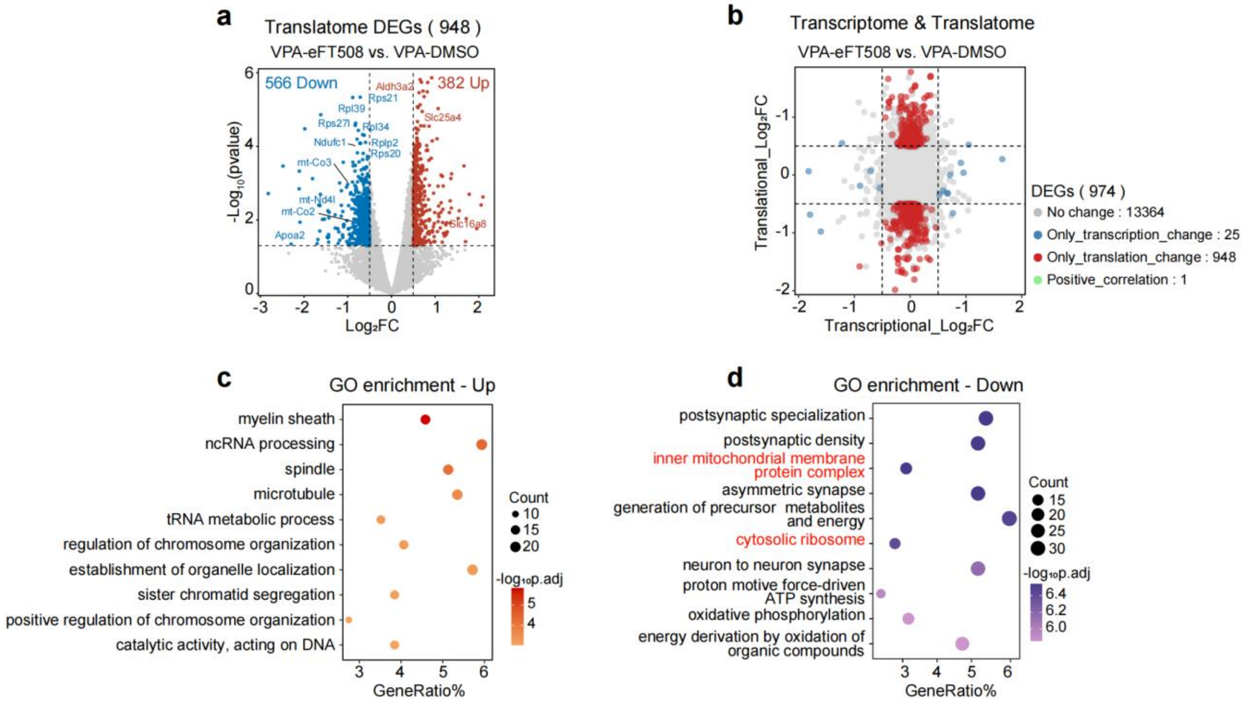

Figure S10. Translatome analysis of the cerebral cortex after administration of eFT508 in VPA mice.

(**a**) Volcano plot showing translational DEGs in the cerebral cortex of VPA-eFT508 vs. VPA-DMSO groups.

(**b**) Log_2FC of mRNAs (VPA-eFT508 vs. VPA-DMSO) at transcriptional and translational levels.

(**c**,**d**) Bubble plots showing GO enrichment results of upregulated (**c**) and downregulated (**d**) translational DEGs in the cerebral cortex of VPA mice after eFT508 treatment.

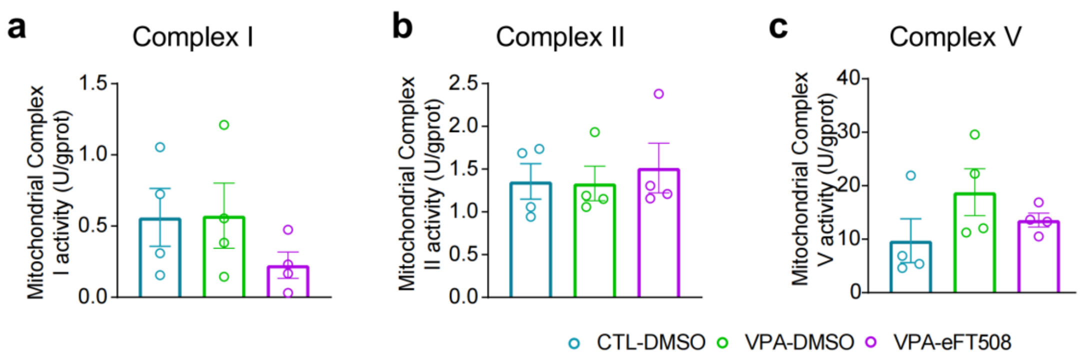

Figure S11. The activities of the mitochondrial Complex I, II and V did not exhibit significant changes after administration of MNK inhibitor eFT508 in cerebral cortex of VPA mice. (**a**–**c**) Mitochondrial Complex I (**a**), Complex II (**b**), and Complex V (**c**) activity in the cerebral cortex of 6-week-old mice of different groups. *n* = 4 mice for each group. One-way ANOVA.

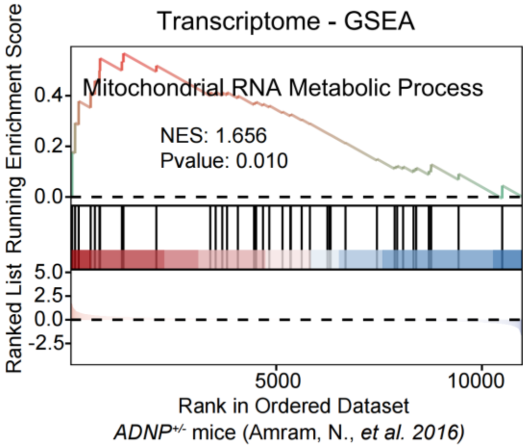

Figure S12. Enhanced mitochondrial RNA metabolic process in male *ADNP*$^{+/-}$ mice.
GSEA showing upregulation of mitochondrial RNA metabolic process (GO term) in the transcriptome datasets from *ADNP*$^{+/-}$ mice reported by Amram, N., *et al* (71).

Supplemental experimental procedures

Elevated Zero-maze

The test mouse was placed in one of the junctional areas of the open and closed sections, facing toward the closed section, of the elevated Zero-maze, and allowed to explore freely for 5 min. The distance traveled in the open and closed sections, the head dip times in the open sections, and the number of crossings to the open sections were analyzed.

Novel Object Recognition Test

The novel object recognition test was conducted in a 40 cm (length) × 40 cm (width) × 40 cm (height) box. Following a 10-minute habituation phase in the empty box, the test mouse was subject to a 10-min training phase, during which two identical objects were placed in the box. The test mouse was allowed to explore these objects for 10 minutes. One hour after the training phase, one of the identical objects was replaced with a novel object of a different shape. The test mouse was then allowed to explore both the familiar and novel objects for 10 minutes. 24 hours after the training phase, one of the identical objects was replaced with another novel object of a different shape. The test mouse was allowed to explore both the familiar and novel objects for 10 minutes. The total travel distance of the test mouse and the sniffing time spent on different objects were analyzed during each phase.

Y-maze test

The Y-maze test was conducted in a Y-shaped maze composed of three identical arms, each measuring 34 cm (length) × 8 cm (width) × 14 cm (height), spaced 120° apart. The test mouse was placed at the end of one arm randomly, and its sequence of arm entries was recorded over an 8-minute period. An entry was defined as the mouse having all four limbs within the arm. A correct alternation was defined as the mouse entering three different arms consecutively. The spontaneous alternation rate was calculated using the following formula: Spontaneous alternation rate = Number of correct alternations/(Times of total arm entries−2)×100%.

Barnes Maze Test

The Barnes maze test was conducted over 5-day trials on a circular platform with 20 holes equally

spaced around the perimeter, one of which (the target hole) led to a black escape box. The location of the target hole was consistent for a given mouse but randomized across mice. After the last training session, a probe test was conducted without the escape box for 3 min. The time spent around each hole was recorded.

Table S1. Translational DEGs list (VPA vs. CTL)

Table S2. Expressions of core regulators of mRNA Poly(A) tail modification (VPA vs. CTL)

Table S3. GO analysis of translational DEGs (VPA vs. CTL)

Table S4. GSEA analysis for translatome data (VPA vs. CTL)

Table S5. Syndromic gene list (n = 464) from SFARI database

Table S6. List of Genes with ≥ 10 supporting reports (n = 472) from SFARI database

Table S7. Translational DEGs list (VPA-eFT508 vs. VPA-DMSO)

Table S8. eFT508 reversed translational DEGs list (changed in VPA vs. CTL but reversed in VPA-eFT508 vs. VPA-DMSO)

Table S9. GO analysis of eFT508 reversed translational DEGs (changed in VPA vs. CTL but reversed in VPA-eFT508 vs. VPA-DMSO)

Table S10. Statistical Details
